# Single‐cell third‐generation sequencing‐based multi‐omics uncovers gene expression changes governed by ecDNA and structural variants in cancer cells

**DOI:** 10.1002/ctm2.1351

**Published:** 2023-07-30

**Authors:** Lei Chang, Enze Deng, Jun Wang, Wei Zhou, Jian Ao, Rong Liu, Dan Su, Xiaoying Fan

**Affiliations:** ^1^ GMU‐GIBH Joint School of Life Sciences Guangdong‐Hong Kong‐Macau Joint Laboratory for Cell Fate Regulation and Diseases Guangzhou National Laboratory Guangzhou Medical University Guangzhou Guangdong Province P. R. China; ^2^ Innovation centre for Advanced Interdisciplinary Medicine The Fifth Affiliated Hospital of Guangzhou Medical University Guangzhou Guangdong Province P. R. China; ^3^ Department of Biomedical Devices The Bioland Laboratory (Guangzhou Regenerative Medicine and Health Guangdong Laboratory) Guangzhou Guangdong Province P. R. China; ^4^ Present address: Department of Cellular and Molecular Medicine University of California San Diego La Jolla California USA; ^5^ The Guangzhou Institutes of Biomedicine and Health Chinese Academy of Sciences Guangzhou Guangdong Province P. R. China

## Abstract

**Background:**

Cancer cells often exhibit large‐scale genomic variations, such as circular extrachromosomal DNA (ecDNA) and structural variants (SVs), which have been highly correlated with the initiation and progression of cancer. Currently, no adequate method exists to unveil how these variations regulate gene expression in heterogeneous cancer cell populations at a single‐cell resolution.

**Methods:**

Here, we developed a single‐cell multi‐omics sequencing method, scGTP‐seq, to analyse ecDNA and SVs using long‐read sequencing technologies.

**Results and Conclusions:**

We demonstrated that our method can efficiently detect ecDNA and SVs and illustrated how these variations affect transcriptomic changes in various cell lines. Finally, we applied and validated this method in a clinical sample of hepatocellular carcinoma (HCC), demonstrating a feasible way to monitor the evolution of ecDNA and SVs during cancer progression.

## Graphical abstract

scGTP‐seq is a third‐generation sequencing (TGS) platform‐based single‐cell multi‐omics method that enables simultaneous sequencing of the genome and transcriptome. This method can efficiently detect ecDNA and SVs and determine how these genomic variations impact transcriptomes at the single‐cell level. It is a powerful tool to investigate the regulatory mechanisms of ecDNA and SVs across diverse cell types and clinical tumour samples.

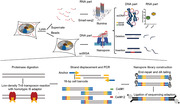

## 1 INTRODUCTION

In 1965, extrachromosomal DNA (ecDNA) fragments were reported for the first time in neuroblastoma and paediatric brain tumour cells.^1^, ^2^ In recent years, a growing number of studies have reported that ecDNA is abundantly present in cancer and senescent cells, suggesting its involvement in the initiation and development of tumours and senescence.^3^, ^4^, ^5^, ^6^ Pan‐cancer research showed that ecDNA can be effectively detected in many human cancer types with abundant variation, and confirmed that it is highly correlated with poor clinical outcomes.^7^, ^8^ It has been proposed that oncogenes and drug‐resistant genes can be amplified with ecDNA replication, leading to an increase in gene expression, which contributes to the development and evolution of cancer cells.^4^, ^9^ In addition, efficient replication and asymmetric partitioning of ecDNA to two daughter cells serve as potential mechanisms of tumour heterogeneity and evolution.^4^, ^10^, ^11^, ^12^ In 2019, Wu and colleagues investigated how the structure of ecDNA affected oncogene expression independently of copy number amplification, as ecDNA‐derived oncogene expression was found to be higher than that on linear chromosomes after normalizing with copy number.^13^ ATAC‐seq and Hi‐C sequencing further showed that ecDNAs lack the higher‐order compaction that is characteristic of chromosomal DNA, display significantly enhanced chromatin accessibility and interacts more often with active chromatin regions. These results suggested that the loose compaction of ecDNA and its sub‐nuclear localization close to active chromatin regions are associated with the enhanced expression of ecDNA‐carried genes.^13^ Other studies reported that enhancer elements included in the ecDNA structure are involved in the transcriptional regulation of ecDNA genes^14^ and chromosomal oncogenes.^15^ Moreover, genome remodelling caused by the reintegration of ecDNA structures could have profound effects on gene expression including the disruption of tumour suppressor genes and the augmentation of oncogenes.^16^ The circular recombination of ecDNA in seismic amplification can further promote genomic amplification and evolution in cancer cells.^9^ Another study demonstrated that the novel mutagenesis of ecDNA was mediated by APOBEC3.^17^ Hung et al. showed protein‐tethered ecDNA hubs and delineated their roles in promoting intermolecular enhancer–gene interactions and oncogene overexpression.^18^ A CRISPR‐based live‐cell imaging method, ecTag, was developed to identify uneven segregation of ecDNA and transcriptionally active ecDNA hubs in cancer cells.^19^ However, to date, efficient methods to reveal how ecDNA regulates gene expression in heterogeneous cancer cell populations at single‐cell resolution have not been reported.

The emergence of single‐cell sequencing technology during the past decade has made it possible to investigate heterogeneous cell populations with unprecedented resolution, and to unveil previously hidden details from the averaged signal obtained from bulk sequencing analysis.^20^, ^21^, ^22^ Single‐cell sequencing can be used to study genomic sequences,^23^, ^24^, ^25^ DNA methylome,^26^, ^27^ chromatin accessibility,^28^, ^29^, ^30^ transcriptome,^31^, ^32^, ^33^, ^34^ and histone modifications or transcription factor binding.^29^, ^35^, ^36^, ^37^, ^38^, ^39^, ^40^, ^41^, ^42^ However, most single‐cell sequencing approaches are based on next‐generation sequencing (NGS) platforms that are typically biased for library fragments shorter than 1 kb. Recently, the development of third‐generation sequencing (TGS) has overcome the limitations associated with short‐read lengths obtained from NGS.^43^ For example, it is difficult to identify the splicing variants of transcripts from short reads. However, in recent years, TGS‐based single‐cell RNA sequencing (scRNA‐seq) methods have been developed to accurately identify alternative splicing events from full‐length transcripts.^44^, ^45^ Previous NGS‐based single‐cell whole‐genome sequencing (scWGS) enabled an investigation of the heterogeneity of copy number variation (CNV) and single‐nucleotide variation, allowing the analysis of cell lineage tracing. However, due to the length limitations of NGS sequencing reads, these scWGS methods are not suitable to analyse large‐scale variations at the genomic level, such as structural variants (SVs) and ecDNA, that are frequently observed in various diseases. Conversely, the long reads obtained from the TGS platform serve as a promising feature that enables the identification of SVs and ecDNA. The TGS platform nanopore has been used in ecDNA sequencing, both at the bulk level,^18^ and after isolation with a novel targeted ecDNA purification method, CRISPR‐CATCH.^46^ In addition, TGS‐based scWGS methods have recently been developed that permit the identification of SVs and ecDNA.^47^

Single‐cell multi‐omics technologies have enabled the integration of information from multiple sequencing modalities, and have facilitated the establishment of a more accurate correlation between genotype (genomic sequence, DNA methylome and chromatin accessibility) and phenotype (transcriptome and proteome).^48^ According to the central dogma,^49^ genomic variation can lead to transcriptional variation and underlie phenotypic plasticity among single cells. Multi‐omics analysis helps not only to investigate the heterogeneity of cells within a tissue, but also to depict the intertwined regulatory relationships between different omics layers. Moreover, cell lineage trajectories reconstructed by multi‐omics can identify the causal mutations that regulate the transition between cellular states.^50^, ^51^, ^52^ In addition, the integration of data from multi‐omics can enhance the accuracy of variant calling, which is crucial, for example, to the diagnostic screening of blastomeres.^53^ In 2014, the first method that co‐detected DNA and RNA from a single cell was developed,^54^ which was followed by genomic DNA and mRNA sequencing,^55^ Genome & Transcriptome sequencing,^56^ the simultaneous isolation of genomic DNA and total RNA,^57^ and TARGET‐seq.^58^ These studies consistently found a positive correlation between CNVs and transcription levels. This suggests that once the genomic patterns of SVs/ecDNA mutations and transcriptome are simultaneously analysed in the same single cell, it will be possible to precisely determine how different genomic mutations influence the downstream phenotype.

Here, we developed a novel multi‐omics method termed single‐cell paralleled genome and transcriptome sequencing on a third‐generation platform (scGTP‐seq). To achieve the co‐detection of genomic DNA and mRNA, we physically separated the transcriptome and the genome, then modified the SMOOTH‐seq procedure^47^ with paralleled scRNA‐seq analysis using Smart‐seq2.^59^ We also developed the ecDNAFinder pipeline to facilitate scGTP‐seq data analysis, and confirmed that it can efficiently identify ecDNAs from TGS reads. The ecDNAs in different cancer cell types showed cell‐type specificity. Specifically, the ecDNAs were abundantly detected in certain genomic regions with high copy numbers, suggesting ecDNA hotspots. Moreover, the same ecDNA found in different cell types showed distinct abundance, while different ecDNA genes exhibited variable copy numbers in the same cells. Although the ecDNA genes showed globally higher expression levels, this variation does not linearly correlate with the ecDNA copy number. We further analysed whether SVs induced downstream transcriptome alterations and identified multiple deletion and insertion events that led to altered transcripts. Finally, we applied scGTP‐seq on a clinical hepatocellular carcinoma (HCC) sample, which contained multiple cell types, and identified ecDNA from both cancer cells and non‐cancer cells. Thus, scGTP‐seq can serve as a powerful tool to investigate the effects of ecDNA and SVs in heterogeneous cancer samples.

## 2 RESULTS

### 2.1 Design and optimization of scGTP‐seq

**FIGURE 1:**
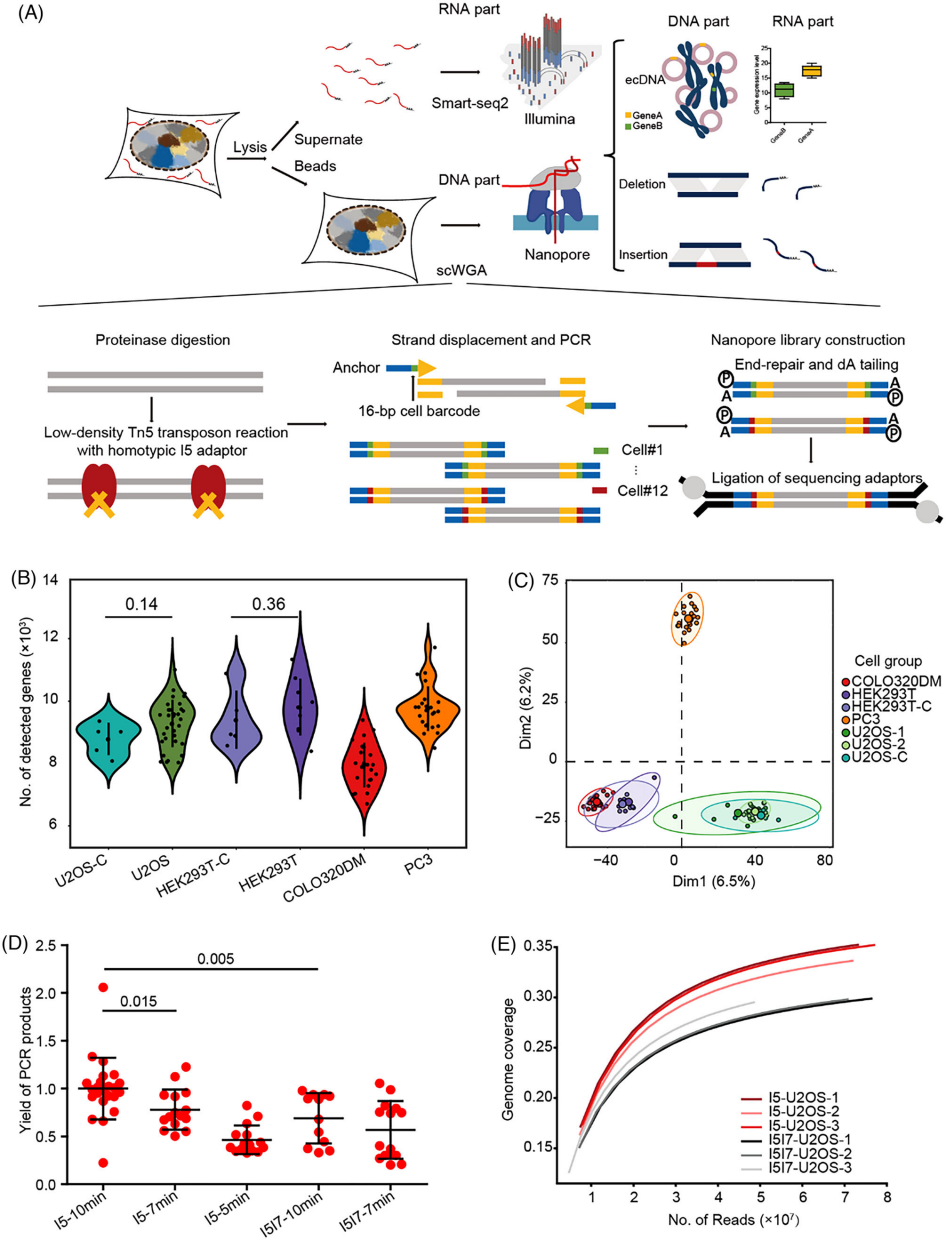
Development of scGTP‐seq.

In design, we used carboxylic acid magnetic beads to separate DNA and RNA molecules in each single cell to obtain the DNA and RNA separately for downstream amplification and sequencing. The magnetic beads can accumulate around the cell surface to hold the cell in the pellet, while the RNA was released to the permeabilisation buffer in the supernatant (Supporting Information Figure S1A). Then, Smart‐seq2 was applied to amplify the RNA in the cell lysate. Subsequently, the adjusted TGS‐based scWGS method, SMOOTH‐seq^47^ procedure and library construction for TGS were performed with the genomic DNA attached to the magnetic beads (Figure 1A). We applied scGTP‐seq in four cultured cell lines, including human embryonic kidney cell line HEK293T, osteosarcoma cell line U2OS, colon cancer cell line COLO320DM and prostate cancer cell line PC3, to validate the robustness of the method. First, we evaluated the sensitivity and accuracy of transcript detection in different single cells. For the majority of cell types, the average number of detected genes per cell is approximately 9000, except for COLO320DM cells, which is 7951 (Figure 1B and Supporting Information Table S1). To examine whether separating the genomic DNA in scGTP‐seq interfered with transcriptome detection, we then analysed the RNA modality of U2OS cells and HEK293T cells generated by scGTP‐seq, and compared to that scRNA‐seq profiles of same cell lines generated using whole‐cell Smart‐seq2. We found no significant difference between the number of detected genes in both cell lines (Figure 1B), suggesting that scGTP‐seq has comparable RNA detection sensitivity with whole‐cell Smart‐seq2. Furthermore, principal component analysis (PCA) and unsupervised clustering revealed that the cells belonging to the same type clustered together. Both U2OS and HEK293T cells matched well to the corresponding whole‐cell Smart‐seq2 controls (Figure 1C and Supporting Information Figure S1B), suggesting no detectable bias in scGTP‐seq. In addition, the U2OS cells that were performed in different experimental batches showed no difference on both the PCA plot and the cluster dendrogram (Figure 1C and Supporting Information Figure S1B), indicating that the scGTP‐seq workflow is highly reproducible. In summary, the transcriptome data of scGTP‐seq exhibit advanced sensitivity, accuracy and stability in different cell types.

The initial amount of DNA required for TGS library construction is approximately at the microgram level, and the length of the DNA fragments should reach several kilobases. Thus, the current scWGA methods are not suitable for TGS analysis. Hence, we addressed the demands of scWGS on the TGS platform based on the SMOOTH‐seq method, with modifications to facilitate the amplification of separated DNA on magnetic beads (Figure 1A). Different combinations of Tn5 adaptors, as well as transposase reaction duration, were systematically tested to examine the resulted length and yield of genomic amplicons. The results showed that, when compared to the heterotypic I5I7 adaptor, longer genomic fragments were obtained using the Tn5 enzyme loaded with the homotypic I5 adaptor. This implementation also improved the yield of the PCR amplification product, which may be due to the fact that only 50% of the library molecules contain two different adaptors at each end of the molecule that can be amplified by the heterotypic I5I7 adaptor PCR^60^ (Figure 1D and Supporting Information Figure S2A). Further sequencing revealed that the genome coverage obtained with the homotypic adaptor was higher than that achieved with the heterotypic adaptors (Figure 1E). These suggest a better genome capture efficiency with Tn5 transposase loaded with the homotypic adaptor sequences.

The isolated single‐cell DNA was tagmented with Tn5 for a time series ranging from 5 to 10 min. The DNA fragments obtained by a 5‐min Tn5 treatment were larger in length than those achieved by 7‐ and 10‐min treatments, which showed similar length distributions (Supporting Information Figure S2B). However, the amplification yield of fragments obtained from the 5‐min Tn5 treatment was the lowest, which may be due to the difficulty in amplifying large DNA fragments with PCR (Figure 1D). Even though no essential difference was observed in the size distributions of the DNA fragments obtained from 7‐ or 10‐min Tn5 treatment, the latter resulted in a significantly higher amplification yield (Figure 1D). In addition, we evaluated the amplification bias by calculating the genome coverage at different read depths and compared the results from cells amplified using our method with published data using other methods.^61^ Our single‐cell DNA amplification method (scGTP‐seq cell#1, scGTP‐seq cell#2) outperformed other single‐cell DNA amplification methods including MALBAC, MDA and eWGA (Supporting Information Figure S2C). Taken together, we decided to use homotypic I5 adaptor‐loaded Tn5 transposases to treat the single‐cell DNA isolated with the magnetic beads for 10 min in scGTP‐seq to achieve a high yield of long DNA amplicons.

Considering the high cost of using scWGS on the TGS platform, we decided to first conduct the scRNA‐seq analysis, followed by scWGS analysis for single cells that showed high scRNA‐seq data quality. Accordingly, the single‐cell DNA attached to the magnetic beads has to be stored for a certain amount of time, which could potentially affect the quality of the subsequent scWGS data. We experimentally confirmed that sample storage in −80°C for 4 weeks did not impair scWGS data quality. To our surprise, the frozen samples outperformed the fresh ones in terms of DNA library yield and read mapping ratio. In addition, the frozen samples showed significantly higher genome coverage (Supporting Information Figure S2D). These data suggest that scGTP‐seq could be adapted in practical settings with excellent robustness.

### 2.2 Analysis and verification of ecDNA identified by scGTP‐seq

The DNA amplicons of each cell contained a 16‐bp cell barcode sequence that is compatible with TGS platforms. Twelve single cells were pooled together considering the sequencing cost and the requirement of initial DNA amount for TGS library construction. Nanopore and PacBio are two representative TGS sequencing platforms utilizing different principles. As SMOOTH‐seq has only been tested on the PacBio platform, we further compared sequencing results from both technologies, specifically the Nanopore PromethION and the PacBio sequel II with HiFi mode using 12 cells in a library. The reads obtained from both platforms showed over 98% mapping ratios in individual cells, with PacBio data showing slightly better stability (Supporting Information Figure S3A). However, single cells sequenced on the Nanopore platform had a higher number of mapped reads (Supporting Information Figure S3B). The averaged read lengths varied among different cell types, which were likely caused by variable enzymatic activity of Tn5 transposase when applied to different genomes (Supporting Information Figure S3C,D). For U2OS cells, much longer read lengths were obtained with the Nanopore sequencing, which may lead to a relatively lower mapped read ratio (Supporting Information Figure S3A). The scWGS data acquired from the Nanopore platform exhibited enhanced genome coverage due to its higher read output (Supporting Information Figure S3E,F), thereby offering better capability in identifying structural genomic variations. The error rate from the Nanopore platform is much higher than the PacBio platform (Supporting Information Figure S3G), which could potentially affect ecDNA and SVs identification. Besides, there was a positive correlation between read length and error rate, indicating longer reads may contain more sequencing errors (Supporting Information Figure S3C,G).

**FIGURE 2:**
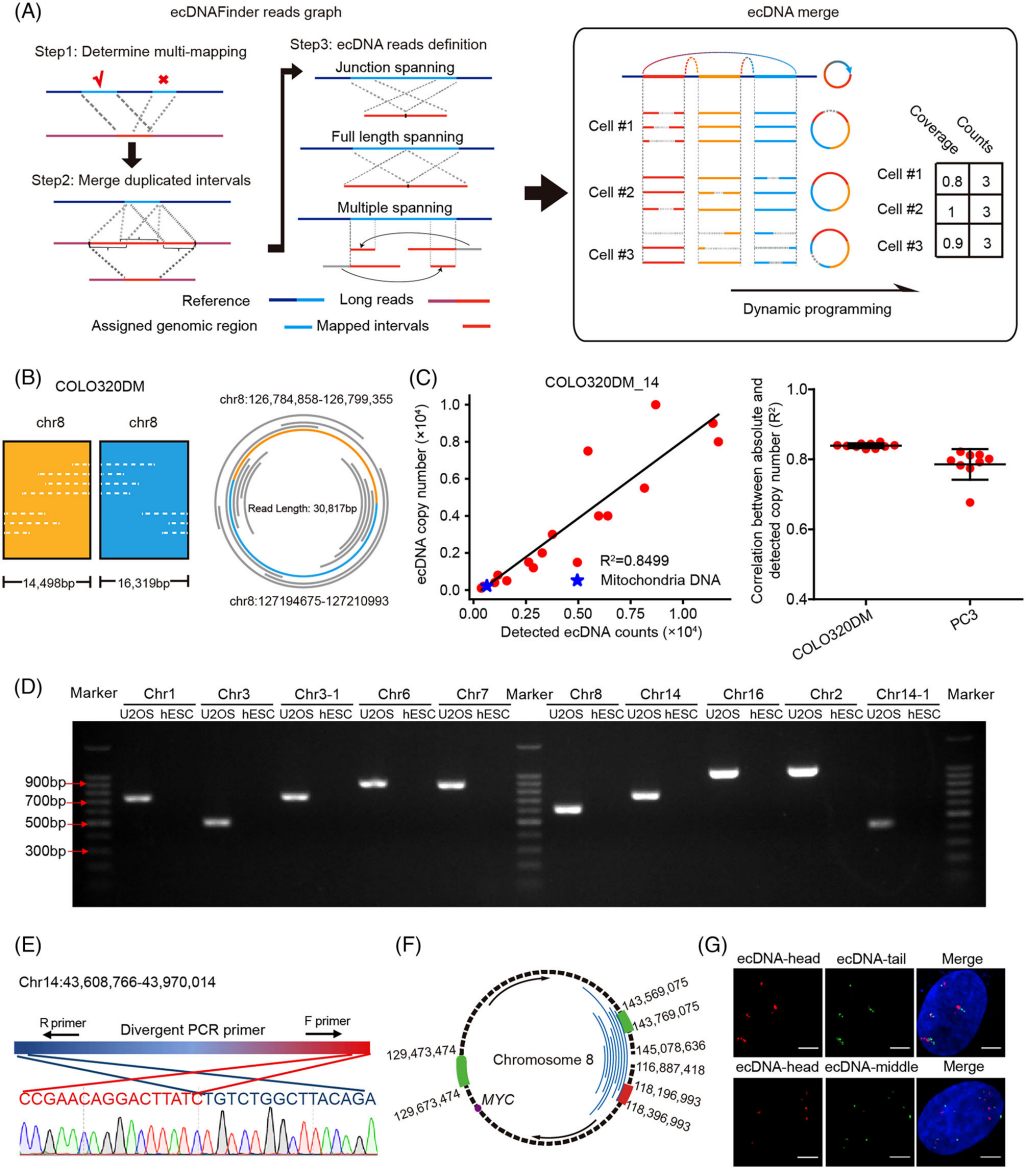
Analysis and verification of ecDNAs.

We next tried to find ecDNA using the scGTP‐seq data. Previous studies have developed analytical tools to resolve ecDNA using WGS reads from NGS platforms.^7^, ^62^ However, few software tools exist for the identification of ecDNA from TGS reads. To address this, we established a bioinformatics pipeline named ecDNAFinder (Figure 2A and Supporting Information Figure S4, Methods). Briefly, multiple aligned read intervals were assigned to the most consistent genomic regions, and secondary mapping reports within the largest mapped interval were removed. The short, duplicated sequences within the reads were merged as they might be generated by sequencing errors. Afterwards, reads that contained separate intervals assigned by at least two reversely located mapped genomic regions were extracted as candidate ecDNA spanning reads. Stringent filtering criteria were adopted to remove false positives. SVs and tandem repeats can generate chimeric reads, which would be identified as false‐positive ecDNA by our ecDNAFinder pipeline. Thus, we removed the candidate ecDNA whose cyclization site appeared within 500 bp‐distance of any SVs identified by Sniffles (including deletions, insertions, translocations, etc., see Methods),^63^ as well as the candidate ecDNA whose 300 bp sequences flanking the cyclization site overlapped with simple tandem repeats for at least 30 bp. In addition, random chimeric reads can also be generated by PCR amplification. To remove these artifacts, we filtered ecDNA that had less than three de‐duplicated support reads with the rationale that the likelihood that random junctions happened in the same position over three times in a PCR reaction is very low. After filtering, the ecDNA identified in single cells was merged according to their genomic coordinates of cyclization sites. The ecDNA type was determined by how many genomic fragments were detected within one ecDNA. Only ecDNA spanning reads were used to quantify the abundance of the corresponding ecDNA in each cell, and the read coverage for each ecDNA in single cells was also calculated. This algorithm allowed us to obtain information regarding the ecDNA types, the chromatin intervals, the coordinates of cyclization sites, the number of supported reads in single cells, the number of supported cells in each cell line and the located genes for each ecDNA (Supporting Information Table S2). Compared with short NGS reads, the several‐kilobases long reads obtained from TGS platform can give more accurate sequence and assemble information of ecDNA. For large ecDNAs, the scGTP‐seq reads could only cover a small region flanking the cyclization sites. The short ecDNAs, including mitochondria DNA (mtDNA), could be fully covered by a single or multiple scGTP‐seq reads (Figure 2B and Supporting Information Figure S5A‐D). Besides, both single and multiple fragments ecDNAs can be identified by ecDNAFinder pipeline (Figure 2B and Supporting Information Figure S5A‐E).

To validate the efficiency of scGTP‐seq methods and ecDNAFinder pipeline, 16 types of circular plasmids (range from 3 to 27 kb long) were added to each scGTP‐seq reaction. Each plasmid was quantified via digital PCR and pooled together according to a concentration gradient (Supporting Information Table S3). The midpoint of the unique region of each plasmid was defined as the cyclization site, and ecDNAFinder enabled the detection of all the types of incorporated plasmids. Moreover, the number of ecDNA reads for the plasmid showed a positive correlation with the absolute plasmid copy numbers (Figure 2C and Supporting Information S6). The high correlation of the measured counts and the actual copy numbers (averaged *R* ^2^ = 0.8) indicated that scGTP‐seq is able to efficiently capture circular DNAs and that can be accurately identified by ecDNAFinder (Figure 2C and Supporting Information Figure S6). Additionally, it was also possible to recover the mtDNA present in each cell (Figure 2C and Supporting Information Figure S6). We quantified the copy number of mtDNA in different cell types. The results showed that the mtDNA copy number varied among different cell types (Supporting Information Figure S5E).

While a higher number of mappable reads were obtained from each single cell using Nanopore sequencing, this did not imply that more authentic ecDNA could be found with Nanopore sequencing reads due to its higher error rate. Hence, a comparison was performed between scGTP‐seq data from U2OS cells using both Nanopore and PacBio platforms. We found that Nanopore sequencing identified five times more ecDNAs compared to PacBio sequencing, with 59% of the ecDNAs found using PacBio sequencing also detected by Nanopore (Supporting Information Figure S7A). Next, 13 ecDNA candidates were chosen from both datasets for validation. We designed divergent PCR primers at both ends of the putative ecDNA region and conducted outward PCR amplification using genomic DNA from U2OS and human embryonic stem cells (hESCs) as templates. The results showed that 10/10 of the ecDNAs identified with Nanopore sequencing data were amplified with expected sizes (Figure 2D and Supporting Information Table S4). Sanger sequencing results also confirmed the candidate cyclization sequences (Figure 2E and Supporting Information Figure S7B). None of the three ecDNA candidates supported only by the PacBio platform could be validated via PCR or Sanger sequencing (data not shown). According to these results, the Nanopore platform outperforms PacBio HiFi sequencing for ecDNA identification in scGTP‐seq, likely because the Nanopore platform enabled higher genome coverage and generated a higher number of longer reads that could be mapped to the genome. Thus, in the following work, we mainly used Nanopore data to build ecDNA profile.

To validate that ecDNAFinder pipeline‐derived ecDNA candidates are bona fide ecDNA, we labelled the oncogene *MYC‐* located ecDNA in U2OS cells with fluorescence in situ hybridization (FISH) probe tiling on 200 kb sequences in the head, tail and middle parts of the ecDNA to verify its circular structure (Figure 2F). Considering U2OS is a cultured cell line that might have an altered karyotype, we labelled non‐ecDNA gene *AC019257.8* on chr8 as a karyotype reference. FISH assay revealed an average of 20 copies of *MYC* gene and only two copies of *AC019257.8* gene within one nucleus (Supporting Information Figure S7C), as well as about 10 copies of each labelled *MYC* ecDNA fragments (Figure 2G), suggesting that these genomic regions were co‐amplified within the ecDNA. Moreover, the distance between the head and tail fragments of the ecDNA was shorter than that between the head and middle fragments, demonstrating the circular structure of the ecDNA (Figure 2G). Overall, the results showed that the scGTP‐seq workflow combined with the ecDNAFinder pipeline enabled the identification of endogenous ecDNAs.

### 2.3 Characterization of ecDNA in different types of cells

**FIGURE 3:**
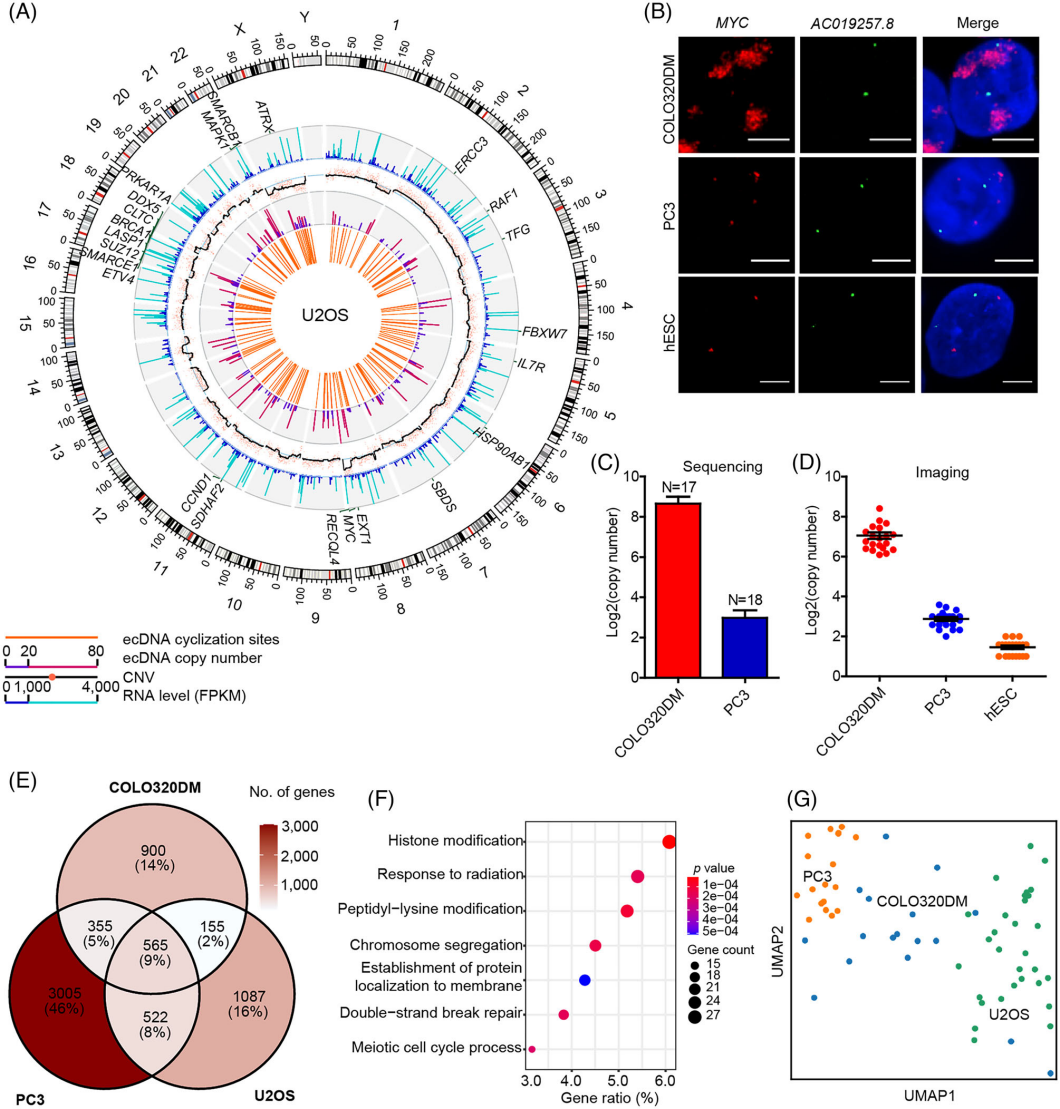
Characterisation of ecDNAs in cancer cell lines.

We constructed ecDNA profiles of U2OS, COLO320DM and PC3 cells using scGTP‐seq data analysed by the ecDNAFinder pipeline. In each cell line, the ecDNA cyclization sites and their respective abundance were summarized in a circular map of the whole genome (Figure 3A, Supporting Information S8A and S8B). To evaluate whether ecDNAs have a potential impact on genetic and transcriptomic features of the cell, the genome copy numbers and the gene expression levels were also analysed using scGTP‐seq data. In all the analysed cell types, the majority of the ecDNAs ranged from 3 to 10 Mb (Supporting Information Table S2). Generally, ecDNA regions showed elevated genome copy number. For example, abundant ecDNAs were derived from chr8 q23.11, chr13 q14.2 and chr16 q21 in COLO320DM cells, where more than two copies of the genome can be detected from these regions (Supporting Information Figure S7A). As for PC3 cells, the chromosomal regions chr5 q23.1‐q23.2, chr10 q22.2 and chr14 q24.3 contained a large number of ecDNAs and increased copy number (Supporting Information Figure S8B). U2OS cells contained an increased copy number of chr8 q23.1 and chr22 q11.22‐q11.23, which might also be caused by the presence of ecDNAs within these regions (Figure 3A). Moreover, we found numerous ecDNAs within the region overlapping the *MYC* gene in all the three cell types, especially in COLO320DM cells. This indicates that there might be some ecDNA hotspots in cancer cells. Using a non‐ecDNA gene *AC019257.8* on chr8 as a karyotype reference, we co‐labelled the *MYC* and the *AC019257.8* genes in the three cancer cell lines along with hESCs (Figure 3B, Supporting Information Figures S7C and S9A), and found a significantly higher number of *MYC* gene copies in COLO320DM cells compared to the PC3 cell lines, which was consistent with the results from the sequencing analysis (Figure 3B‐D). In contrast, there was no increase in *MYC* gene copy number in hESCs.

Even though the ecDNA landscapes of the three cancer cell types were not the same, we identified several ecDNA hotspots, such as the oncogene *MYC*, *CDK4* and *CCND1* genomic loci (Figure 3A, Supporting Information Figure S8A,B). The genes located on ecDNAs in each cancer cell line were all enriched in cancer‐related functions (Supporting Information Figure S9B‐D). There were 565 ecDNA genes present in all three cell types (Figure 3E), whose functions were related to cell proliferation and metabolism including histone modification, response to radiation, peptidyl‐lysine modification and chromosome segregation, etc. (Figure 3F). Furthermore, the differences in ecDNA gene profiles of the three cancer cell lines can provide enough molecular evidence at a single‐cell level to discriminate different cell types (Figure 3G), as cells within the same population showed more similar ecDNA patterns than across different populations. We also generated a catalogue for cell line‐specific cancer genes on ecDNA (Supporting Information Table S5).

### 2.4 Gene expression alterations governed by ecDNA and SVs

**FIGURE 4:**
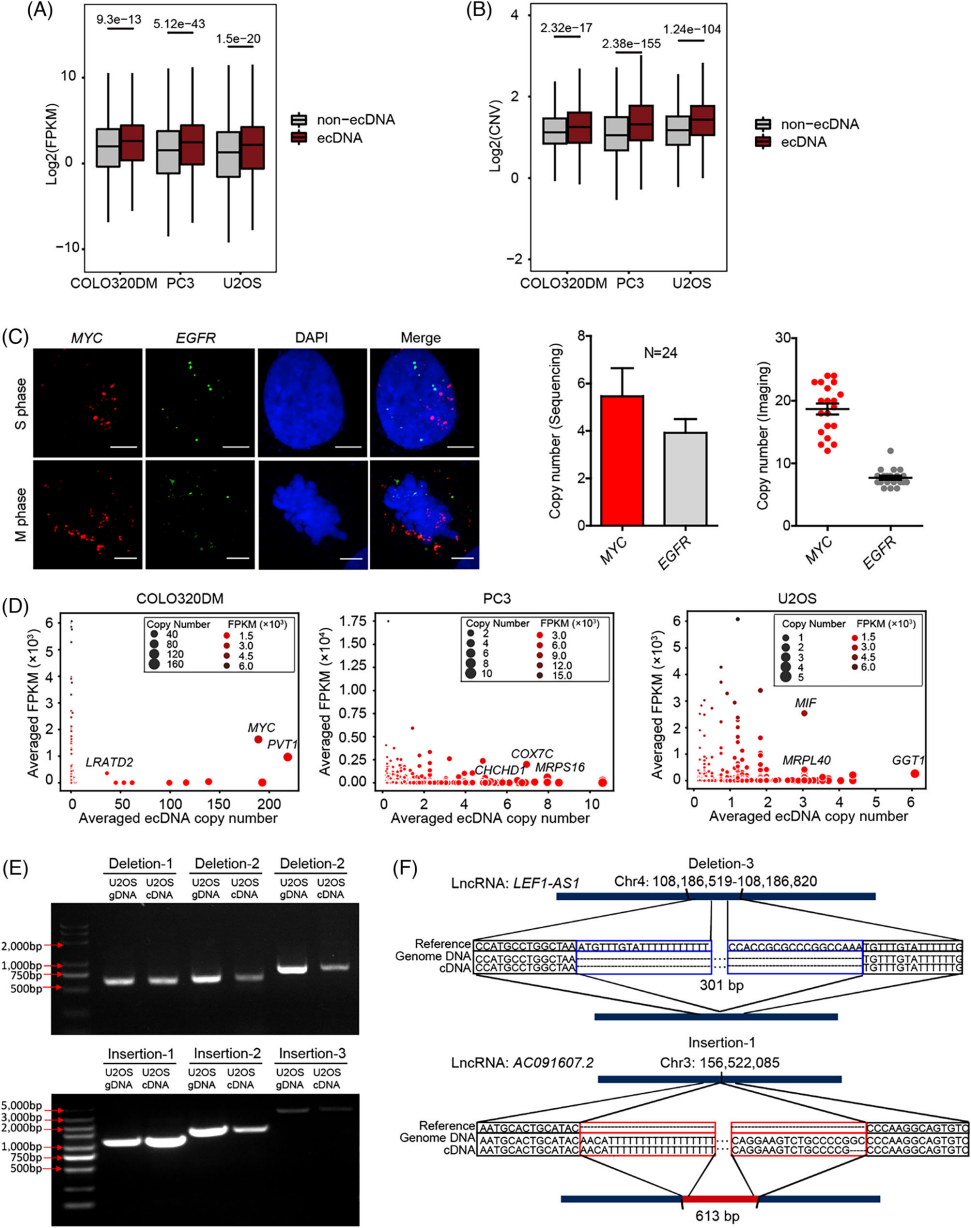
Gene expression and CNV alterations.

In a previous study, Wu and colleagues^13^ reported that genes located on ecDNAs are the most highly expressed genes in cancer cell lines and TCGA clinical tumour samples, as ecDNA can serve as additional DNA template for transcription. Taking advantages of our parallel genome and transcriptome sequencing method in single cells, we first compared the expression levels of ecDNA genes and non‐ecDNA genes in each cell type. As expected, the ecDNA genes had a significantly higher expression level than non‐ecDNA genes (Figure 4A). We further interrogated our data and assessed the correlation between CNV and ecDNA. Genomic regions enriched for ecDNA also showed increased copy number (Figure 4B). In addition, some genomic regions showed enrichment for CNVs but did not have identifiable ecDNA (Supporting Information Table S6). We labelled two ecDNA genes by Oligopaint DNA FISH and found that the abundance of different ecDNA genes varied including *EGFR* and *MYC* in U2OS cells. The observations from imaging showed a higher copy number of the *MYC* gene than the *EGFR* gene, consistent with the sequencing results (Figure 4C). During metaphase, the signal points of *EGFR* and *MYC* genes were partially distanced from the condensed chromosome, which was expected given the extrachromosomal properties of ecDNAs (Figure 4C). To further evaluate how the copy number of ecDNA affects the expression level of the relevant genes, we measured the correlation between copy number and expression level of ecDNA genes (Figure 4D) and found that it were not simply positively correlated. Of note, some ecDNA genes with low copy number had higher expression levels, which indicated the existence of other regulatory mechanisms enhancing the transcription of these ecDNA genes. In addition, an inverse relationship where high‐copy number genes had low expression levels was also found. Together, our data suggest that the copy number may not be the only factor that determines the transcription level of ecDNA genes.

Similar to the identification of ecDNA, long sequencing reads generated from the TGS platform should also facilitate the analysis of SVs. To characterize the SVs from our scGTP‐seq data, we used Sniffles^63^ to identify deletion, insertion and translocation events, and analysed the correlation between these SVs and gene expression changes using the scWGS‐seq data and scRNA‐seq data, both generated with scGTP‐seq of U2OS cells. To remove the potential false‐positives produced by TGS sequencing errors, SVs found in less than three supporting reads were filtered. This strategy enabled the identification of 1082 deletions, 557 insertions and 1782 translocation events from U2OS cells (Supporting Information Figure S10A and Table S7), including some insertions and translocation events on mtDNA (Supporting Information Figure S10B and Table S7). We subsequently mapped the scRNA‐seq reads to the genes containing SVs (Methods), and found that a total of 277 insertions and 292 deletions were transcribed (but no translocations). The genes with transcribed variant RNA molecules with more than two supporting cells for deletions and three cells for insertions were labelled on the SVs circos map (Supporting Information Figure S10A). We further experimentally validated six deletion and insertion events by amplifying these SVs using both the genomic DNA and the cDNA of U2OS cells. Both types of SVs were amplified with the expected fragment lengths using genomic DNA and cDNA (Figure 4E and Supporting Information Table S8). Sanger sequencing of the amplified products further confirmed that the inferred gene structure variation led to transcript variation (Figure 4F and Supporting Information S10C). Together, scGTP‐seq allowed us to identify transcriptomic changes governed by SVs in the genome.

### 2.5 ecDNAs largely exist in HCC cells as well as non‐cancer cells, as revealed by scGTP‐seq

Compared with in vitro cultured cancer cell lines, applying scGTP‐seq to the clinical tumour tissues can be much more challenging, as they have extremely high heterogeneity and need to undergo a long procedure that includes delivery, hard digestion and even sorting, prior to subsequent single‐cell amplification and library construction experiments. HCC is the predominant type of liver cancer, which is one of the top 10 most commonly, diagnosed tumours, and the third leading cause of cancer death worldwide in 2020.^64^ Currently, systematic profiling of ecDNA in HCC is still lacking. To evaluate the performance of scGTP‐seq on discovering ecDNAs from in vivo samples, we applied this method to investigate whether ecDNA is involved in HCC.

**FIGURE 5:**
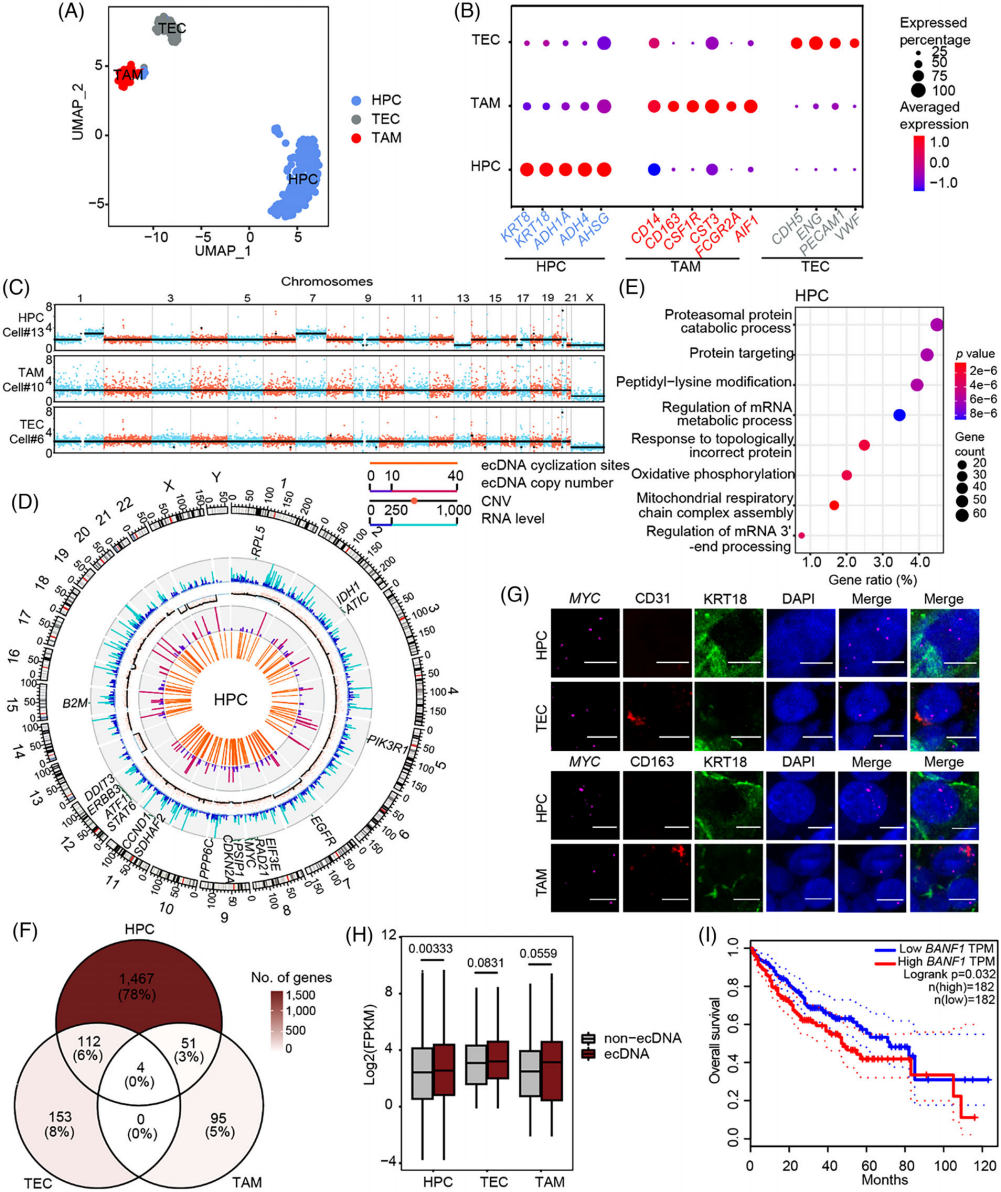
ecDNAs identified in HCC.

The expense of the TGS platform is much higher than that of NGS, which has limited us from analysing a large number of cells via scGTP‐seq. To find the most representative cells in each cell types, we optimized scGTP‐seq by using STRT‐based method^65^ to conduct scRNA‐seq, which can improve the throughput for transcriptome analysis. First, we verified that the gene number detected through STRT is comparable to Smart‐seq2 in U2OS, COLO320DM and PC3 cell lines from 6000 to 12 000 (Supporting Information Figure S11A). In addition, dimensional reduction and clustering analysis revealed that the cells belonging to the same type clustered together on the tSNE plot (Supporting Information Figure S11B). The transcriptome of 384 single cells from a HCC tumour tissue were sequenced via STRT‐based method. After filtering out low quality cells, we obtained single‐cell transcriptome profiles of 319 cells, with an average of 2772 genes detected from each single cell (Supporting Information Figure S11C). These cells could be clustered into three cell types with different gene signatures (Figure 5A and Supporting Information Figure S11D): hepatic cells (HPCs) were characterized by expression of *KRT8*, *KRT18*, *ADH1A*, *ADH4* and *AHSG*; tumour‐associated macrophages (TAMs), characterized by expression of *CD14*, *CD163*, *CSF1R*, *CST3*, *FCGR2A* and *LYZ;* and tumour‐associated endothelial cells (TECs), characterized by expression of *CDH5*, *ENG*, *PECAM1* and *VWF* (Figure 5B). Among these three cell types, HPCs population contained mainly cancer cells, as *KRT8* and *KRT18* were the marker genes of epithelial cells. The gene ontology (GO) analysis of differentially expressed genes in HPCs showed that they were enriched in cell catabolic and metabolic processes, which were highly related with cancer genes (Supporting Information Figure S11E). The inferred CNV obtained from RNA‐seq data also indicated that HPCs had a significantly higher CNV level than the other two cell types, consistent with the characteristic cancer feature (Supporting Information Figure S11F). We further picked 28 HPCs, 8 TECs and 12 TAMs to perform scGTP‐seq. A total of 26 HPCs, 5 TECs and 9 TAMs were kept after removing cells with abnormal CNV patterns. The scGTP‐seq data revealed expected CNV events in HPCs and the non‐cancer cells detected with normal diploid chromosomes (Figure 5C).

Subsequently, ecDNAs were analysed in each cell type. In the HPCs, a total of 139 ecDNAs were found, carrying 51 cancer genes including the genes found within previously described ecDNA such as *MYC* and *CCND1* (Figure 5D). The GO analysis revealed that these ecDNA genes were associated with common processes in cancer, including proteasomal protein catabolic process, protein targeting, peptidyl‐lysine modification and regulation of mRNA metabolic process, etc. (Figure 5E). Surprisingly, we also identified 42 ecDNAs carrying 5 cancer genes in the TECs (Supporting Information Figure S12A) and 47 ecDNAs carrying 1 cancer genes in the TAMs (Supporting Information Figure S12B). Each cell type had specific cancer genes on ecDNA (Supporting Information Table S9). More than half of ecDNA genes in TAMs and TECs were specific (Supporting Information Figure 5F). Next, we selected the *MYC* gene, which was only presented on ecDNAs in HPCs, for experimental verification using FISH (Figure 5G). The copy number of the *MYC* gene was higher in part of HPCs compared to both TECs and TAMs (Figure 5G). We also labelled *CCND1* gene, which appeared in both HPCs and TECs as an ecDNA gene, to verify the authenticity of ecDNAs found in TECs. Indeed, the copy number of *CCND1* significantly increased in TECs compared to TAMs, and also had abnormal increase in some HPCs (Supporting Information Figure S12C,D). This result validated that the ecDNAs we detected in TECs were bona fide ecDNAs. In addition, the ecDNA genes showed significantly higher expression levels than non‐ecDNA genes in HPCs, which was consistent with the results from our previously characterized cancer cell lines (Figure 5H). Furthermore, the ecDNAs were closely correlated to tumour progression. Among the most highly expressing ecDNA genes, we found some had a high correlation with the overall survival rate in HCC, for example, *TRMT112* and *BANF1* (Figure 5I and Supporting Information Figure S12E). Patients with low expression of these ecDNA genes showed a significantly enhanced overall survival probability. The multifunctional methyltransferase subunit TRM112‐like protein, encoded by the *TRMT112* gene, acts as an activator of both rRNA/tRNA and protein methyltransferases.^66^ Barrier‐to‐autointegration factor encoded by *BANF1* is a nonspecific DNA‐binding protein that plays key roles in mitotic nuclear reassembly, chromatin organization, DNA damage response, gene expression and intrinsic immunity against foreign DNA.^67^, ^68^ Both of them have not been characterized as oncogenes. Together, these results indicate that the ecDNA genes identified from scGTP‐seq can serve as new biomarkers for cancer diagnosis and prognosis.

## 3 DISCUSSION

The optimization of existing single‐cell sequencing methods requires improving the sensitivity, accuracy, and coverage of different approaches. The development of new single‐cell multi‐omics methods integrates multiple omics layers and enables comprehensive analysis and precise depiction of cellular heterogeneity. Moreover, these methods provide new approaches to study how different omics layers interplay with each other to determine the fate or status of different cells. In recent years, many single‐cell multi‐omics methods have been developed, allowing investigation on how different genomic features, such as CNV, DNA methylation,^69^ chromatin accessibility,^70^ or histone modifications,^71^ might affect gene expression. The scGTP‐seq first incorporates the TGS platform in single‐cell multi‐omics analysis, offering a novel strategy to study genomic structural variation, including ecDNA and SVs in single cells, and determine how this variation causes downstream changes in the phenotype, as revealed by transcriptional changes. We found that genomic regions found on ecDNAs had higher expression and copy number than non‐ecDNA regions. We have observed that while the differential trends remain statistically significant, the effect sizes appear relatively lower. This phenomenon can be attributed to the inherent heterogeneity of ecDNA within individual cells, as well as our limited sample size. In addition, the relationship between ecDNA copy number and expression level was complex. Some ecDNA genes with low copy numbers still had high expression levels, while high copy number ecDNA genes sometimes had low expression. Our data suggested that copy number might not be the only factor that determined gene transcription level in ecDNA.

The detection of ecDNA using computational tools and WGS data obtained from NGS is quite challenging due to short‐read lengths that require deep sequencing.^72^ Short reads also make it extremely difficult to capture more than one breakpoint at a time.^4^ Compared to WGS, the circle‐seq method can achieve circular DNA enrichment through the digestion of linear genomic DNA and rolling circle amplification, which reduces the requirement for sequencing depth.^16^, ^73^ However, circle‐seq results in the loss of information in linear DNAs. If an ecDNA experiences DNA damage and loses its circular structure, it will not be captured by circle‐seq. However, our scGTP‐seq method is still capable of detecting linearly damaged ecDNA, because the identification of ecDNA candidates relies on the detection of circularization sites. The characteristic long sequencing reads of scGTP‐seq and SMOOTH‐seq, such as the 5–10 kb sequence reads obtained from Nanopore or PacBio sequencing platforms, are more likely to capture the cyclization sites of ecDNA and to provide more conclusive evidence regarding ecDNA frequency and structure.^4^ The long reads obtained from the TGS platform can also achieve higher sequencing coverage of ecDNA and benefit the detection of multiple fragments of ecDNA. Moreover, for relatively short ecDNA (usually no more than 10 kb), a single sequencing read can capture its entire sequence. Although our pipeline can utilize specific sequence features of circularization breakpoints to distinguish ecDNA from most SV events, the distinction between ecDNA and tandem duplication events can pose challenges, especially when working with short reads (Supporting Information Figure S13). Our method, which generates long sequencing reads with an average length of 6 kb, can help reduce false positives by directly identifying short tandem repeats within a single read. Nevertheless, our method may still encounter difficulties in distinguishing ecDNA from large duplication events, especially in long ecDNA candidates that can only be detected through circularization sites. To minimize false positives, we filtered out ecDNAs that overlapped with genome regions annotated as tandem repeats. Additionally, we used DNA FISH as an orthogonal approach to validate our ecDNA candidates.

Furthermore, using long reads of scGTP‐seq, we attempted to investigate potential instances of ecDNA reintegration into the genome. However, none of such events were captured in our data, possibly due to the relatively low sensitivity of ecDNA detection at the single‐cell level. Additionally, the low throughput and high cost of TGS hindered us from deeply sequencing the genome for each individual cell. Understanding the specific processes involved in the generation and integration of ecDNAs, as well as their interactions with chromosomes, is undoubtedly intriguing. However, current sequencing methods can only provide us with limited candidates of ecDNA, which is still crucial but leaves more molecular mechanisms to be explored in future studies.

Currently, the high sequencing cost and limited read output of TGS platforms make them difficult to apply in high‐throughput single‐cell sequencing. Thus, we optimized scGTP‐seq by using STRT‐based method to improve the throughput of the RNA modality. After identifying cell types for each cell, we can choose the cells with high‐quality transcriptome sequencing data from each cell type of interest and process their corresponding scWGS. Besides, the sequencing depth of scWGS is also constrained by low reads output of TGS platforms and limits the detection sensitivity of ecDNA and SVs in scGTP, which means low copy number ecDNAs can be missed. The sequencing cost for each cell in the Nanopore platform is almost half that of the PacBio platform. Nanopore platform can also generate more reads for each cell. However, the relatively high sequencing error rate of TGS platforms, especially for the Nanopore platform, may induce false positives. This may be particularly problematic in the case of very lengthy sequencing amplicons, as longer reads may induce more errors. For example, the U2OS cells sequenced by the Nanopore platform had the longest read lengths but a significantly lower reads mapping rate (Supporting Information Figure S3). With the relatively high errors of Nanopore sequencing, we do not recommend scGTP‐seq for the identification of single nucleotide mutations, but only for detecting large‐scale genetic variation. To remove the false positives induced by sequencing errors as thoroughly as possible, we set stringent criteria in ecDNA reads defining, correction and filtering to find the real ecDNAs using ecDNAFinder, which can further improve the accuracy of ecDNA identification but decrease the sensitivity for low copy ecDNAs. In addition, even though long reads sequenced by TGS platforms increased ecDNA coverage, most of ecDNA we detected still had low coverage, which can benefit from further improvement of TGS platforms in reads length and sequencing depth. In summary, decreases in sequencing cost and sequencing error rate, and increases in sequencing reads output and read length of TGS platform can improve the performance of scGTP in detecting ecDNA and SVs.

Structural variants of the genome, which involve kilobase‐ to megabase‐sized deletions, duplications, insertions, inversions and translocations, represent a major source of genetic variability in somatic cells.^74^ These variants can lead to substantial genetic heterogeneity and contribute to disease development and variable responses to therapy.^75^ According to our scGTP‐seq results, only a tiny fraction of SVs occurred within genes. While insertions and deletions may produce changes in transcript structures, it remains unclear whether these variants lead to changes in protein function or gene regulation. In addition, we did not capture any fusion transcripts generated by translocation events. This may be explained by chimeric RNAs being generated during the post‐transcriptional process such as from splicing.^76^, ^77^, ^78^

Previous studies emphasized the importance of ecDNA in cancer. However, all these analyses were conducted on sequencing data generated from bulk tissue. Thus, it was difficult to pinpoint how ecDNA affected each cell type in heterogeneous cancer tissues. According to our scGTP‐seq results of the HCC tissue, ecDNAs are heterogeneous within a cell population. Moreover, they not only appear in cancer cells, but can also be detected in some non‐cancer cells such as TECs and TAMs. In previous studies, the existence of circular DNA elements in healthy human somatic tissue was verified through circle‐Seq at the bulk level, with these elements considered common mutational components in human soma.^79^ It also mentioned that gene products from ecDNA transcripts could potentially contribute to the phenotype of somatic cells and tissue, as reported in yeast. In light of this, we speculate that the ecDNA detected in TAM and TEC might represent accumulated genomic structural mutations. The stimuli present in the tumour microenvironment could select TAM and TEC that carry specific ecDNA. In our study, only 319 final cells were analysed from a complex cancer tissue, which was restricted to analyse more different cell types within the tissue. Thus, more studies are needed to further elucidate the role of ecDNA in different non‐cancer cell types. We believe that the decrease of TGS sequencing price and the incorporation of automated liquid handling robots in the future will greatly increase the sample size that can be studied using our method.

In conclusion, scGTP‐seq serves as a powerful single‐cell multi‐omic tool to study ecDNA biology, as our results demonstrated that it is feasible to precisely map ecDNAs in different cell types and clinical tumour samples, and obtain deeper insights into ecDNA biogenesis and its roles in cancer progression and evolution.

## 4 MATERIALS AND METHODS

### 4.1 Cell culture

Human osteosarcoma cell line U2OS, prostate cancer cell line PC3, colon cancer cell line COLO320DM and embryonic kidney cell line HEK293T were purchased from the Cell Bank of Chinese Academy of Sciences. U2OS and HEK293T cell lines were cultured in Dulbecco's modified Eagle medium (DMEM) with high glucose (Thermo Fisher Scientific) supplemented with 10% foetal bovine serum (FBS) (ES cell qualified, VISTECH), 1% penicillin/streptomycin (Thermo Fisher Scientific) and 1% Glutamine (Thermo Fisher Scientific). The PC3 cell line was cultured in F12K medium (Thermo Fisher Scientific) supplemented with 10% FBS, 1% penicillin/streptomycin and 1% glutamine. The COLO320DM cell line was cultured in RPMI 1640 medium (Thermo Fisher Scientific) supplemented with 10% FBS, 1% penicillin/streptomycin and 1% glutamine. All the cells were maintained at 37°C and 5% CO_2_ in a humidified incubator.

### 4.2 Single‐cell isolation from the HCC sample

The cancer tissue was handled within 3 h after excision from the patient. Briefly, the tissue was cut into approximately 1 mm^3^ pieces in DMEM, and then enzymatically treated with MACS tumour dissociation kit (Miltenyi Biotec, Cat. 130‐095‐929) using 37C_h_TDK_3 program in the gentleMACS Octo Dissociator with Heaters. Dissociated cells, while re‐suspended in DMEM, were subsequently filtered through a 70 μm cell strainer (BD) and centrifuged at 400 *g* for 10 min at 4°C. After removing the supernatant, the cell pellet was re‐suspended by 1X phosphate‐buffered saline (PBS) with 10% FBS, and the red blood cells were removed using the red blood cell lysis buffer (Roche), according to the manufacturer's instructions. Cells were then filtered through a 40 μm cell strainer (BD), and single cells were picked up by mouth pipetting for scGTP‐seq.

### 4.3 Spike‐in plasmids preparation

Plasmids with lengths between 3,300 and 27,300 bp were chosen and each plasmid had a unique sequence. After extraction from *E. coli*, the plasmids were digested by Exonuclease V (RecBCD, NEB, Cat. M0345) to remove noncircular DNA, and then purified with Ampure XP beads (Beckman, Cat. A63882). The absolute concentration of each plasmid was measured by a Naica crystal digital PCR system (Stilla Technologies). The spike‐in plasmids mixture contained 16 different plasmids with a gradient ratio from 1 to 100 (Supporting Information Table S3).

### 4.4 ScGTP‐seq library preparation and sequencing

After digestion, single cells were placed into 5‐μL cell lysis buffer by mouth pipetting. The cell lysis buffer contained 0.2 μL balanced Dynabeads™ MyOne™ Carboxylic Acid beads (Thermo Fisher Scientific, Cat. 65011), 0.8 U/μL RNase Inhibitor (Takara, Cat. 2313B), 0.2% Triton X‐100 (Sigma‐Aldrich, Cat. X100), 2 μL 5X SuperScript II first‐strand buffer (Invitrogen), 0.4 μM reverse transcription (RT) primer (AAGCAGTGGTATCAACGCAGAGTACTTTTTTTTTTTTTTTTTTTTTTTTT), 10 nM DTT and 0.04% Tween‐20. The single‐cell suspension was vortexed thoroughly for 30 s and placed on an ice‐cold magnet rack.

#### 4.4.1 RNA modality

The supernatant containing RNA molecules was transferred to a new tube, incubated at 72°C for 3 min to release the linearized RNA molecules and immediately placed on ice. Then, 5 μL RT mixture was added into the RNA lysate, containing 20U/μL SuperScript II reverse transcriptase (Invitrogen, Cat. 18064071), 0.8U/μL RNase inhibitor, 0.2 μM RT primer, 2 mM dNTP mixture (Thermo Fisher Scientific, Cat. R0193), 2 M betaine (Sigma‐Aldrich, Cat. B0300), 12 mM MgCl_2_ (Sigma‐Aldrich, Cat. 63020) and 2 μM TSO primer (AAGCAGTGGTATCAACGCAGAGTACATrGrG+G, rG represents riboguanosines and +G represents the locked nucleic acid‐modified guanosine). After briefly mixing and centrifugation, the RT reaction was performed in a thermocycler with the following program setting: 25°C for 5 min, 42°C for 60 min, 50°C for 30 min and 70°C for 10 min. Then, a 15 μL PCR mixture containing 12.5 μL 2× KAPA HiFi hot‐start ready mix and 333 nM of ISPCR oligo (AAGCAGTGGTATCAACGCAGAGT) was added into each tube. The cDNA from the single cell was amplified using the following thermal cycling program: 4 cycles of 98°C for 20 s, 65°C for 30 s and 72°C for 3 min, followed by 16 cycles of 98°C for 20 s, 67°C for 15 s and 72°C for 3 min, with a final cycle at 72°C for 5 min. The PCR product from each cell was purified twice with 0.8× Ampure XP beads (Beckman, Cat. A63882), and then proceeded for library construction using the TruePrepTM DNA Library Prep Kit V2 for Illumina (Vazyme, Cat. TD501/TD502/TD503).

For cells digested from HCC tissue, we performed the single‐cell transcriptome amplification following a STRT‐based method as previously described.^65^ PCR products with different barcodes (up to 96) were pooled together and purified with Zymo DNA clean & concentrator kit (Zymo, Cat. D4014). The pooled cDNA product was further purified with 0.6× Ampure XP beads twice. Then a second round of amplification was carried out with the biotinylated primer (/Biotin/CAAGCAGAAGACGGCATACGAGAT[6bpindex]GTGACTGGAGTTCAGACGTGTGCTCTTCCGATC) and the ISPCR oligo for four to six additional cycles. After purification, the cDNA was sheared into approximately 300 bp fragments with a Covaris sonicator (S220). The fragmented cDNA containing barcode sequences was enriched using Dynabeads® MyOne™ Streptavidin C1 beads (Invitrogen, Cat. 65002) and then used for library preparation using KAPA hyper prep kits (KAPA, KK8505). The adapter‐ligated fragments were amplified using the illumina read2 primer (CAAGCAGAAGACGGCATACGA) and the illumina read1 primer (AATGATACGGCGACCACCGAGATCTACACTCTTTCCCTACACGAC). The scRNA‐seq libraries were sequenced on the illumina Novaseq 6000 system with a PE 150 bp sequencing setup.

#### 4.4.2 Genomic DNA modality

The carboxylic acid beads containing genomic DNA were re‐suspended by adding 2.5 μL DNA lysis buffer, containing 10 mM Tris‐EDTA (Sigma, Cat. T9285), 1 mg/mL QIAGEN protease, 0.3% Triton X‐100 and 200 mM KCl. The mixture was incubated at 50°C for 1 h, followed by protease inactivation at 70°C for 30 min, and was stored at −80°C. After confirming the samples for further amplification based on scRNA‐seq data analysis, 7.5 μL tagmentation mixture (13.3 mM TAPS‐NaOH [pH = 8.3], 6.67 mM MgCl_2_, 10.67% PEG8K and 1 μL 0.2 ng/μL adaptor loaded Tn5 enzyme [TruePrep Tagment Enzyme, Vazyme, Cat. S601‐01] diluted in Tn5 storage buffer) was added to cell lysate, mixed gently, and incubated at 55°C for 10 min. The Tn5 tagmentation reaction was stopped by adding 2.5 μL 0.2% SDS (Solarbio, Cat. S1015) and holding at room temperature for 5 min to quench Tn5 activity. The strand displacement of the Tn5 adaptors and amplification of the fragmented genomic DNA was carried out by adding 37.5 μL PCR mixture, composed of 25 μL 2×Gflex PCR Buffer (Mg^2+^, dNTP plus), 0.033 U/μL Tks Gflex DNA polymerase (TAKARA, Cat. R060B) and 1.067 μM I5 PCR primer containing a 16‐bp cell barcode (5′AATGATACGGCGACCACCGAGATCTNNNNNNNNNNNNNNNNTCGTCGGCAGCGTC3′). The amplification of genomic DNA was conducted by the following PCR program: 72°C for 3 min, 98°C for 1 min, 20 cycles of 98°C for 15 s, 60°C for 30 s, 68°C for 5 min and a final cycle at 68°C for 5 min. The genomic DNA amplicons with different barcode sequences were pooled together and purified with 0.8× Ampure XP beads. A total of 400 ng to 1 μg genomic DNA amplified products were used for further library construction and sequenced on the Nanopore PromethION or PacBio sequel II with HiFi mode.

### 4.5 ScRNA‐seq data pre‐processing

Raw paired‐end reads were trimmed using Cutadapt (version 3.3) with parameters ‘–trim‐n ‐q 13,11 ‐n 5 ‐m 20 ‐B/‐b ‘AAGCAGTGGTATCAACGCAGAG;max_error_rate = 0.02’ ‐B/‐b ‘CTCTGCGTTGATACCACTGCTT;max_error_rate = 0.02’. Then the reads were aligned to the hg38 genome reference with STAR (version 2.7) after further quality trimming by TrimGalore (version 0.6.5). The bam files of the same cells from multiple lanes were merged by Samtools (version 1.1) and duplicates were marked by Picard MarkDuplicates (version 2.25.1). We calculated the normalized gene expression levels as FPKM (fragments per kilobase per million mapped reads), which was assessed by RSEM (version 1.3.3) subcommand rsem‐calculate‐expression. The detailed description and code are available online: https://github.com/WellJoea/SmartSeq2Pipe.git

### 4.6 The comparison of gene expression in single cells

The single‐cell expression matrix was normalized by the Seurat (version 3.2.3) built‐in function NormalizeData and scaled by function ScaleData with default parameters. Then PCA was conducted and visualized by R package factoextra (version 1.0.7) using default parameters. Pearson's correlation coefficients were calculated using normalized expression levels for all the cells to examine transcriptome similarity between different cell lines and different batches characterized by scGTP‐seq. Similar samples were grouped by hierarchical clustering analysis using distance ‘one minus Pearson correlation coefficients’ by function heatmap.2 from R package gplots.

### 4.7 The RNA analysis of HCC samples

The analysis of HCC followed the Seurat (version 3.2.3) standard workflow. Single‐cell gene expression profiles were filtered when gene number was less than 300, UMI number was larger than 20 000, or mitochondrial gene percentage was higher than 20%. K‐nearest neighbours analysis was performed based on 30 principal components and then clustering was conducted by ‘louvain’ method with resolution of 0.5. Cell types were identified based on expression of marker genes. The genes enriched in each cell type were found by ‘FindAllMarker’ function in Seurat package with log fold‐change cut‐off = 0.25. The CNV score for each cell was calculated by R package InferCNV using ‘infercnv::run’ function with a cut‐off value of 0.1.

### 4.8 Pre‐processing of single‐cell genome third‐generation sequencing data

PacBio sub‐reads were transformed to consensus reads using CCS (version 4.2.0). Different cells pooled in the same library were separated by de‐multiplexing the cell barcodes using Lima (version 2.0.0). The CCS reads were mapped to the human genome (hg38) using PBMM2 (version 1.4.0).

Nanopore read sequences were called from fast5 files using guppy (Oxford Nanopore Technologies, version 2.3.7). Reads were then trimmed by Cutadapt (version 3.3) with parameters ‘‐q 13,11 ‐m 100’ and aligned using Minimap2 (version 2.17) with the following parameters: ‘‐ax map‐ont ‐t 20 –cs –MD ‐Y ‐L –secondary no’. Reads and mapping quality were, respectively, checked by FastQC (version 0.11.9) before mapping and with Samtools (version 1.1) after mapping.

To evaluate the plasmids sequences in the sequencing data, each circular plasmid was split into a linear reference, and we defined the middle position of the longest unique region as the cyclization site. The 16 plasmids linear reference were added to the hg38 reference genome during mapping and annotation.

### 4.9 CNV analysis with TGS reads

Human hg38 reference genome was downloaded from the UCSC database (https://genome.ucsc.edu/cgi‐bin/hgTables). After excluding the *N*‐gaps region, the hg38 reference genome was split into consecutive bins that were 500 kb in length. A total of 5824 bins (except chromosome Y and mitochondrial chromosome) were obtained, and we further estimated the number of reads primarily and supplementarily aligned within each bin (with at least 10 bp overlap).

After normalizing the total depth of each bin by sequencing data volume, a locally weighted scatterplot smoothing (LOWESS) model was used for GC‐content correction. The copy number of each bin was defined by the ratio of read counts and predicted values of the final model and normalized by log2 transformation. Then, we calculated the copy number and corrected the bias caused by whole genome amplification.

To infer discrete copy number segments, the circular binary segmentation algorithm was adopted in R package DNAcopy. In brief, we transformed the copy number matrix to a CNA object, used smooth CNA function to smooth single‐point outliers and calculated the segmentation with segment function. A gain was defined as a segment with a copy number value > 2.6, and a loss was defined as a segment with a copy number value < 1.4. Only those CNVs > 500 kb were kept for downstream analysis. Code for long reads CNV analysis is available at https://github.com/fanxylab/LongCNV.git.

### 4.10 ecDNA detection by ecDNAFinder

The ecDNA detection started with fetching mapping read intervals and referred to genomic locations. Then we performed multiple steps including: (1) detecting reads rearrangements to get the ecDNA candidate reads; (2) constructing the breakpoint graph for each read; (3) concatenation of consensus fragments; (4) annotating mapped genome coordinates, filtering, and visualization of the circles. Here, we briefly described the implementation of the pipeline, and the full code can be accessed from https://github.com/fanxylab/EcDNAFinder.git. We have provided executable example data on CodeOcean, which can be accessed through the following link: https://codeocean.com/capsule/4396048/tree/v1. In addition, we have packaged the code into a Docker container: https://hub.docker.com/r/fanxylab/ecdnafinder.

After mapping to the reference, only the primary and supplemental alignments were kept, and the corresponding read intervals and mapped genomic coordinates were extracted.

Then we discarded the ‘LOWALIGN’ intervals with a length less than 100 bp, merged the ‘OVER’ intervals which were completely contained in the adjacent interval, and removed the ‘DUPLIC1’ or ‘DUPLIC2’ intervals which reported similar mapped genomic start and end coordinates of the adjacent intervals. Finally, we converted adjacent intervals into breakpoints.

Next, we merged breakpoints when they meet the following three conditions: (1) the two reference genome regions on which the two breakpoints are mapped being located in the same chromosome; (2) in the same mapping orientation; (3) the genomic distance between two breakpoints being less than 500 bp. Then, we linked each breakpoint to the most adjacent breakpoint and constructed a breakpoint graph. The ecDNAs were defined from breakpoint graph including a single‐interval self‐cyclization. To exclude the false positives induced by tandem repeats and SVs in the genome, we filtered out candidate ecDNAs whose 300 bp sequences flanking the cyclization site overlapped with the simple tandem repeats for over 30 bp (http://hgdownload.soe.ucsc.edu/goldenPath/hg38/database/simpleRepeat.txt.gz). We also removed the candidate ecDNA whose cyclization site that appeared within 500 bp‐distance near any SVs including deletion, duplication, insertion, inversion, inversion‐duplication and translocation events detected by Sniffles. The supporting reads number of each ecDNA should be no less than 3.

The same cyclization site may be reported as inconsistent coordinates caused by sequencing errors. The consensus intervals were merged if they were located within 500 bp on both ends. Then we updated the consensus ecDNA based on the longest interval, and the other included intervals were rearranged according to the concensus. Finally, the merged ecDNA list and the supporting reads details were summarized.

### 4.11 ecDNA gene annotation

We kept ecDNAs with at least three supporting reads in the merged data of each cell type. The ecDNA genes were annotated when they overlap with the hg38 annotation gtf file for at least 30 bp. The circles diagram was plotted using circus (version 0.69), providing information of ecDNA distribution, abundance, the corresponding genome CNV and gene expression. The cancer genes were sourced from https://cancer.sanger.ac.uk/cosmic/census,^80^ and a total of 576 Tier 1 genes were chosen without any additional filtering criteria.

### 4.12 Evaluation of ecDNA detection using plasmids

To check if ecDNA detection was consistent with the absolute abundance, we calculated the read counts evaluated by the ecDNAFinder pipeline for each plasmid in single cells, and performed linear regression between the detected numbers and the suggested absolute copy numbers for the plasmids in each individual cell. The *R* ^2^ values from the linear regression analysis were evaluated to assess the correlations.

### 4.13 Comparison of ecDNA from different datasets

We compared the similarity of ecDNA identified in U2OS cells from PacBio sequencing and Nanopore sequencing, in COLO320DM cells and PC3 cells from our data and published data^13^ using pybedtools (version 2.29.1) intersect function, with the arguments ‘s = False, S = False, wa = True, wb = True, f = 0.1, F = 0.1.″

### 4.14 SVs analysis in U2OS cells

We used Sniffles (version 1.0.12) with at least three supporting reads to detected SVs with the scGTP‐seq DNA data of the U2OS cells. The SVs were then filtered according to their lengths and qualities. Here, we mainly focused on three types of SVs: deletions, insertions and translocations, which were applicable to pair‐wise transcriptome analysis. For deletions and insertions, the maximum length cut‐off values were set to 100 and 2.5 kb, respectively. The minimum cut‐off values were all set to 100 bp. Finally, SVs with the quality ‘PRECISE’ were kept.

We noticed that many cells might share the same SV events, that is, certain types of SVs from different cells occurred on the same chromosome and had similar start and end positions. We constrained the similar position as following: in the genome, if we define SV A happens at breakpoint iA and jA and SV B happens at breakpoints iB and jB, A and B could be regarded as the same SV event when (iA‐500) < iB < (iA + 500) and (jA − 500) < jB < (jA + 500). In this way, we merged similar SVs in different cells into one individual SV with relatively a wide range and fuzzy genome positions. The SVs were further annotated with gene, exon and intron information using bedtools v.2.30.0 with the parameter ‘intersect’.

### 4.15 Integrated analysis of transcriptome data and the genome structure alterations

We extracted genes located in ecDNA regions and analysed the expression changes caused by ecDNA in each cancer cell line. Then the gene lists of different cell types were compared and visualized using *R* package ggVennDiagram.

The functional GO analysis of ecDNA genes was conducted by *R* package ClusterProfiler with a *p* value cut‐off of 0.01. Qualified GO terms with top gene ratios were visualized. In addition, similar GO terms were consolidated, with one representative term being kept.

To realign the RNA‐seq data to the genomic variants, we removed SVs whose associated genes were not expressed. For each insertion event, we created a new reference sequence that linearly connected all the insertion sequences within the insertion genomic site. The unmapped RNA‐seq reads were realigned to the new references using BWA (version 0.7.17) with the algorithm BWA‐MEM and default parameters. After realignment, reads with at least 100 bp mapped to insertion regions would be considered as a candidate transcript produced by this insertion structure. Similarly, for one deletion event, 500 bp sequences flanking the genome coordinates of the SVs were connected. If the single end reads could span the breakpoints and cover atleast 100 bp length, we regard them as transcripts generated from deletion events. The translocation events might result in fusion transcripts, and therefore, we detected gene‐fusion events from scRNA‐seq. We ran Star‐Fusion version 1.9.1 on trimmed Fastq files using GRCh38_gencode_v33_CTAT_lib_Apr062020 as the genome library and then summarize fusion transcripts from every single cell. We then compared the locations of translocations and fusion transcripts to check whether they were matchable. Code for SVs analysis and in silicon validation is available at https://github.com/fanxylab/SV.git

### 4.16 Survival analysis on HCC patients

We selected various ecDNA genes and committed overall survival analysis for each of them using a web‐based tool GEPIA (http://gepia.cancer‐pku.cn). Overall survival is defined as the time interval between the date of diagnosis to the date of death from any cause or last follow‐up. LIHC dataset was used for the analysis with default parameters.

### 4.17 Sequencing saturation analysis

We compared the genome capture efficiency between homotypic Tn5 adaptor (I5) and heterotypic adaptor (I5I7) in Figure 1E. The NGS reads of Tn5 transposon‐based scWGA samples were aligned to the hg38 reference genome using BWA (version 0.7.17) with default parameters. The base quality score recalibration (BQSR) was applied for mapping recalibration with GATK (version 4.2.0) BaseRecalibrator and ApplyBQSR commands. We then randomly extracted reads with certain fractions from sorted bam files using DownsampleSam tool from picard.jar (version 2.25.1). The proportions of reads ranged from 0.1 to 0.9 with interval of 0.05. For every fraction, we obtained a new bam file and then assessed the read depths by Samtools depth command line. Next, the number of covered genome bases was calculated per fraction and divided by the length of human genome (bp) to get the genome coverage.

### 4.18 PCR validation of ecDNA and SVs

The ecDNAs and SVs found in U2OS cells were chosen for PCR verification. Genomic DNA of U2OS cells and hESCs were extracted using the DNeasy Blood and Tissue Kit (QIAGEN) following instructions for mammalian cells. The RNA of U2OS cells were extracted using the RNeasy Mini Kit (QIAGEN) following the instructions for mammalian cells, and cDNA was obtained by RT as described previously.^81^ For each ecDNA, we designed a pair of outward PCR primers to identify the cyclization site by primer designing tool in NCBI website (https://www.ncbi.nlm.nih.gov/tools/primer‐blast/, Supporting Information Table S4). For each SVs event, we designed a pair of inward PCR primers to identify the inserted or deleted sequence by the NCBI primer designing tool. The PCR amplification was carried out using Phanta Max Super‐Fidelity DNA Polymerase (Vazyme, P505). The lengths of PCR products ranged from 400 bp to 1 kb, as determined by 1.5% agarose gel electrophoresis. The PCR products on agarose gel were cut and purified for Sanger sequencing to confirm the amplification of the cyclization sites and SVs sites. All the primers used for validation are summarized in Supporting Information Table S8.

### 4.19 DNA FISH

The Oligopaint FISH libraries were chosen from the database (Probes for Human genome, build 38. Mining Settings: ‘Balance’) generated by the Wu Lab (https://oligopaints.hms.harvard.edu/). Probe sets were designed using standard procedures to target the ecDNA localized genes defined in Supporting Information Table S10. For the primary oligo pool, we purchased the Oligoarray pool (Synbio Technologies), and prepared FISH probes as described previously.^82^ The Oligopaint FISH staining procedure was carried out following the previous description.^83^ Images of nuclei were collected with a Dragonfly spinning disc microscope (Andor), processed by Fiji (Fiji Is Just ImageJ, software available at https://imagej.net/Fiji/Downloads), and analysed by Imaris version 8.4.1 (Bitplane Inc., software available at http://www.bitplane.com/).

### 4.20 Tissue slices preparation and immuno‐FISH

The HCC tissue was snap‐frozen in optimal cutting temperature compound and stored at −80°C for several days before cryosectioning into 20‐μm thick slices. Sections were thawed at room temperature. Then the tissue slices were fixed with 4% PFA for 15 min and then permeabilised with 0.5% Triton‐X in PBS for 30 min. Samples were blocked with blocking buffer containing 5% BSA and 0.1% Triton‐X in 1× PBS for 30 min, incubated with primary antibodies and secondary antibodies at room temperature for 1 h, respectively. Antibodies were diluted in blocking buffer. The antibody was removed by washing the samples three times in 1× PBS at room temperature for 5 min each time. Finally, the samples were post‐fixed in 4% PFA for 10 min and then we performed DNA FISH as previously described. For HCC tissue slices immuno‐FISH, rabbit anti‐PECAM1 (Abcam; Ab28364; 1:50) (TEC marker), rabbit anti‐CD163 (Abcam; Ab182422; 1:100) (TAM marker) and mouse anti‐KRT18 (Abcam; Ab668; 1:100) (HPC marker) were used as primary antibodies. Alexa Fluor 488 anti‐mouse IgG (H+L) (Thermo Fisher Scientific; A21202; 1:1000), Alexa Fluor Plus 555 anti‐rabbit IgG (H+L) (Thermo Fisher Scientific; A32732; 1:1000) and Alexa Fluor Plus 647 anti‐rabbit IgG (H+L) (Thermo Fisher Scientific; A32733; 1:1000) were used as secondary antibodies.

## CONFLICT OF INTEREST STATEMENT

Lei Chang, Wei Zhou, Jun Wang, Enze Deng, Jian Ao, Rong Liu, Dan Su and Xiaoying Fan declare that they have no conflict of interest.

## FUNDING INFORMATION

This work was supported by the National Natural Science Foundation of China (32071451 to X.F. and 32101187 to L.C.), the Guangdong Provincial Pearl River Talents Program (2021QN02Y747), and the R&D Program of Guangzhou National Laboratory (ZL‐SRPG2201704).

The code for pre‐processing scRNA‐seq data can be accessed online at https://github.com/WellJoea/SmartSeq2Pipe.git. For long reads CNV analysis, the code is available at https://github.com/fanxylab/LongCNV.git. The code for SVs analysis and *in silico* validation can be found at https://github.com/fanxylab/SV.git. In addition, we have packaged the code into a Docker container: https://hub.docker.com/r/fanxylab/ecdnafinder.

## Supporting information

Supporting Information

Supporting Information

Supporting Information

Supporting Information

Supporting Information

Supporting Information

Supporting Information

Supporting Information

Supporting Information

Supporting Information

Supporting Information

## Data Availability

All raw and processed sequencing data generated in this study have been submitted to the Genome Sequence Archive in National Genomics Data Center, China National Center for Bioinformation/ Beijing Institute of Genomics, Chinese Academy of Sciences (GSA; https://ngdc.cncb.ac.cn/bioproject/browse/PRJCA005009), which are publicly accessible at under accession number PRJCA005009.
