# Supplementary material for: Single‐cell third‐generation sequencing‐based multi‐omics uncovers gene expression changes governed by ecDNA and structural variants in cancer cells
Source: Clin Transl Med. 2023 Jul 30;13(8):e1351. doi: 10.1002/ctm2.1351 (PMC10387328; doi:10.1002/ctm2.1351)
Supplement: Supplementary file 1 — Supporting Information [file CTM2-13-e1351-s002.docx]

**Single-cell third-generation sequencing-based multi-omics uncovers gene expression changes governed by ecDNA and structural variants in cancer cells**

Lei Chang^1,2,3#╂*^, Enze Deng^1,3#^, Jun Wang^1,3#^, Wei Zhou^3#^, Jian Ao^2^, Rong Liu^1,3^, Dan Su^1,3^, Xiaoying Fan^1,2,3,4*^

^1^GMU-GIBH Joint School of Life Sciences, The Guangdong-Hong Kong-Macau Joint Laboratory for Cell Fate Regulation and Diseases, Guangzhou National Laboratory, Guangzhou Medical University, Guangzhou, 510005, Guangdong Province, P.R. China

^2^The Fifth Affiliated Hospital of Guangzhou Medical University, Guangzhou, P.R. China

^3^The Bioland Laboratory (Guangzhou Regenerative Medicine and Health Guangdong Laboratory), Guangzhou, P.R. China

^4^The Guangzhou Institutes of Biomedicine and Health, Chinese Academy of Sciences, Guangzhou, P.R. China

^#^These authors contributed equally: Lei Chang, Enze Deng, Jun Wang and Wei Zhou

^╂^Current address: Department of Cellular and Molecular Medicine, University of California San Diego, La Jolla, CA 92093, USA

^*^Corresponding author: Xiaoying Fan email: [fan_xiaoying@grmh-gdl.cn](mailto:fan_xiaoying@grmh-gdl.cn)

Lei Chang email: lec008@health.ucsd.edu

**Supplemental information
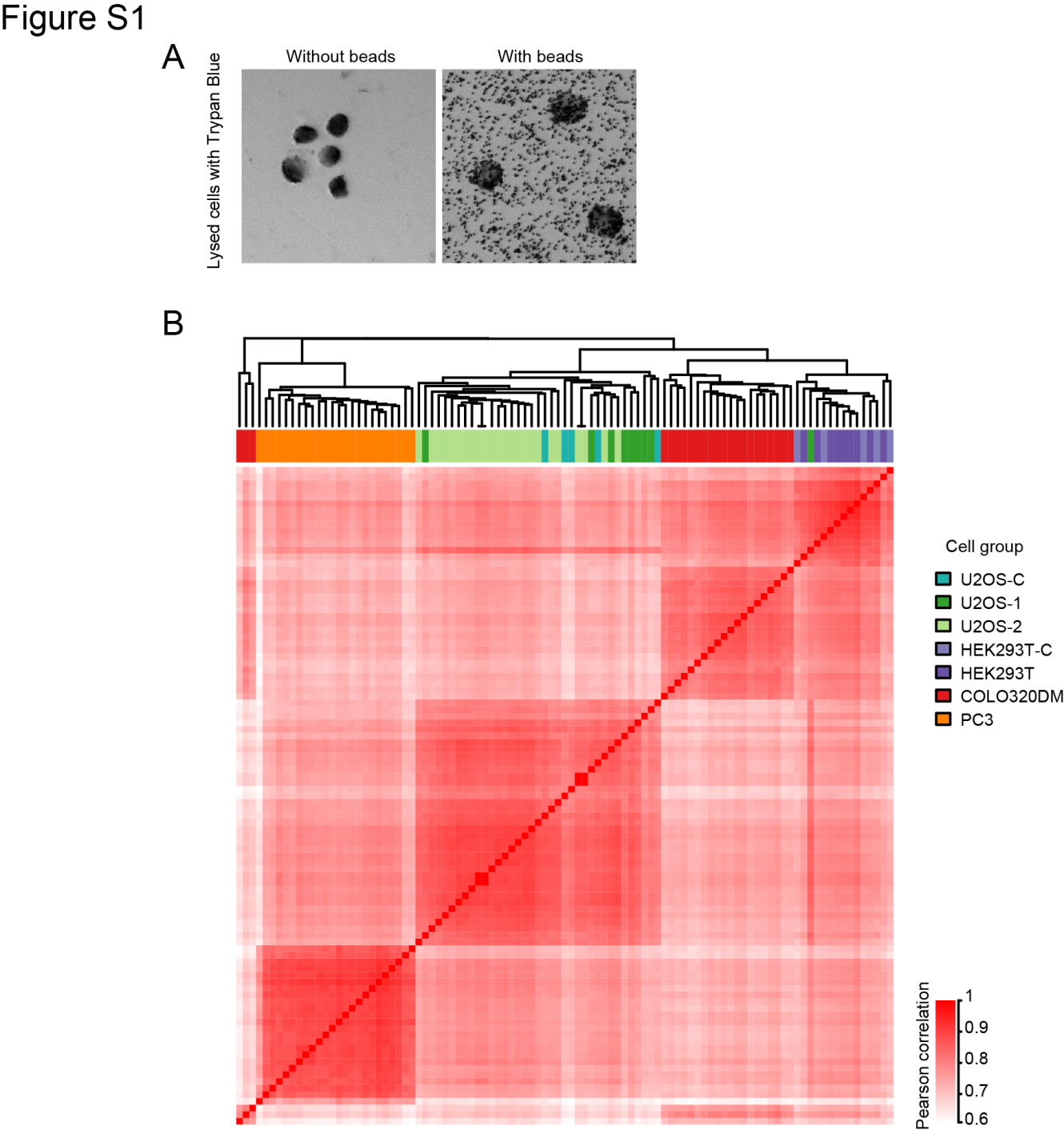
**

**Figure S1.** Development of scGTP-seq.

(A) Using magnetic beads to conduct the DNA isolation in scGTP-seq. Lysed Hela cells were stained by Trypan Blue.

(B) Hierarchical clustering of all cells shown in Figure 1C. The Pearson correlation between each two cells is shown in the heatmap.


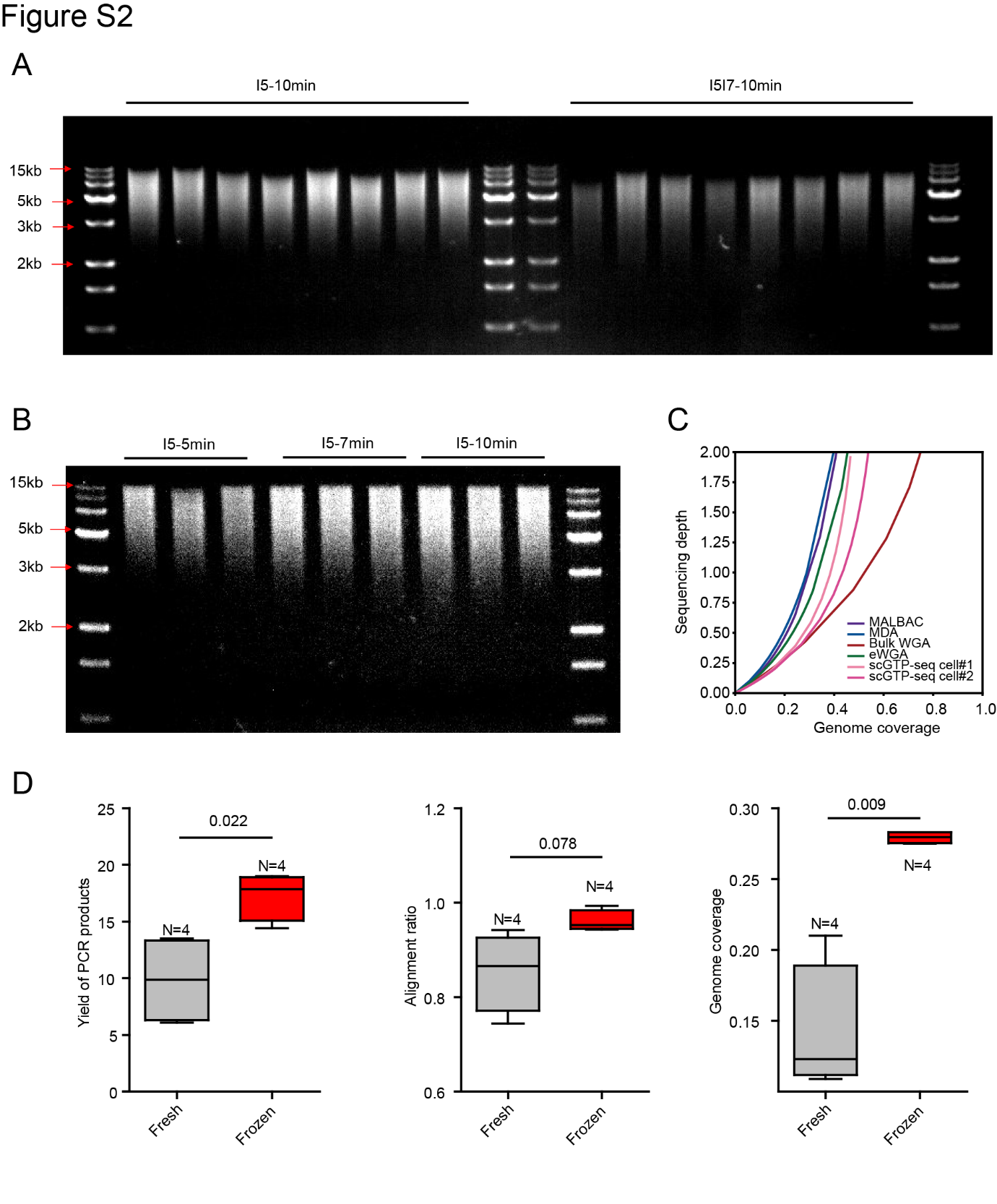


**Figure S2.** Optimization of scGTP-seq process.

(A) Agarose-gel analysis of the PCR products amplified from single-cell genomes treated by Tn5 enzymes loaded with I5 or I5I7 adaptors for 10 minutes.

(B) Agarose-gel analysis of the PCR products amplified from single-cell genomes treated by Tn5 enzymes loaded with I5 adaptor for 5, 7 and 10 minutes.

(C) The comparison between different WGA methods in terms of amplification bias by calculating the genome coverage at different read depths.

(D) The comparison between fresh and frozen single-cell genome samples in terms of PCR product yields, alignment ratio of WGS data obtained from NGS, and their genome coverage with 1.5 million reads. The black lines in the middle of boxplot represent median values, upper and lower ends of boxplot show the upper and lower quartiles, and the whiskers indicate the maximum and minimum values. *p* values are calculated with the two-tailed Student’s *t*-test.


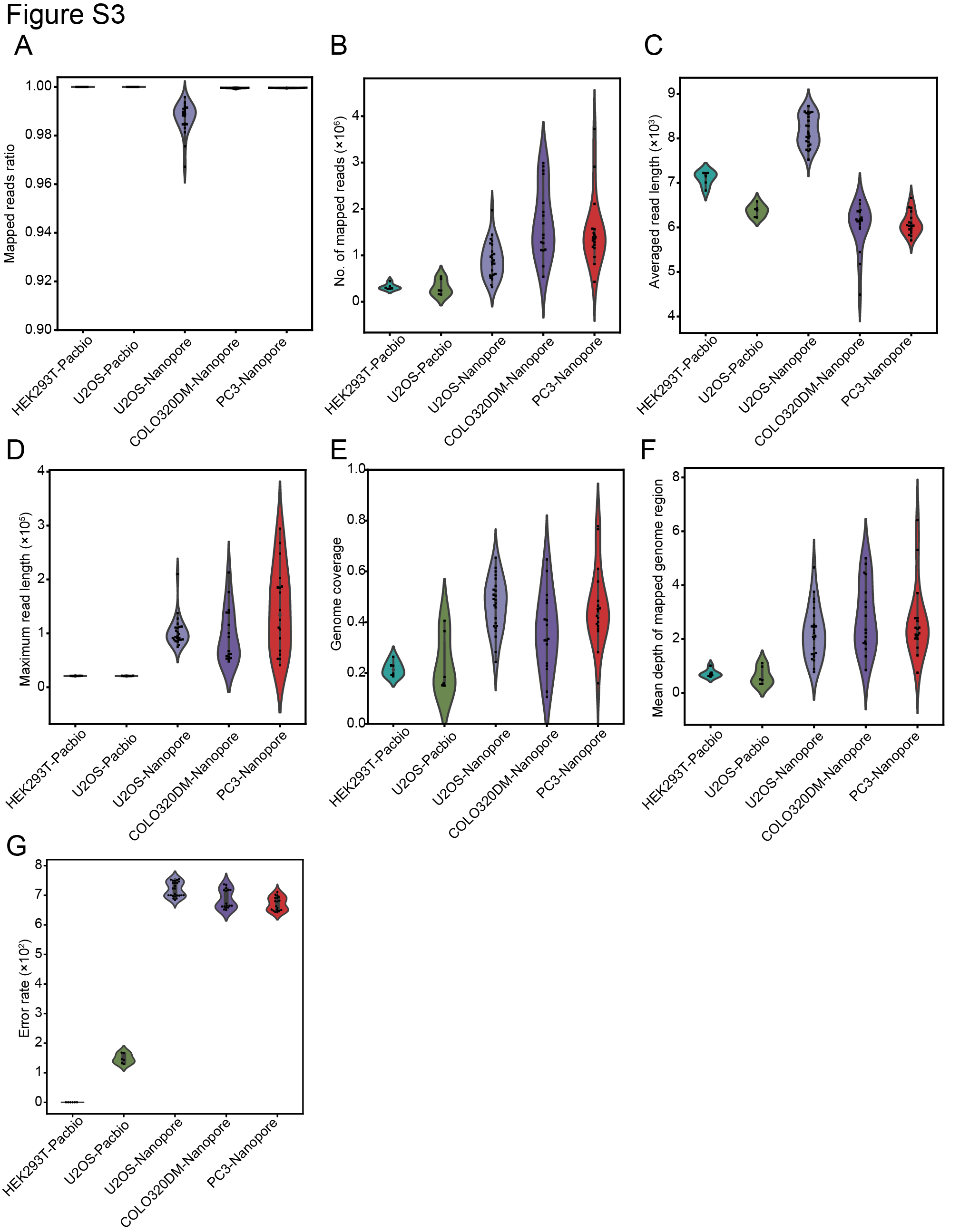


**Figure S3.** The comparison of reads quality between PacBio and Nanopore sequencing data.

The ratio (A), total number (B), average length (C), maximum length (D) of mapped reads, and genome coverage (E), mean depth of mapped genome region (F), and error rate (G) in each single cell are shown as violin plots.


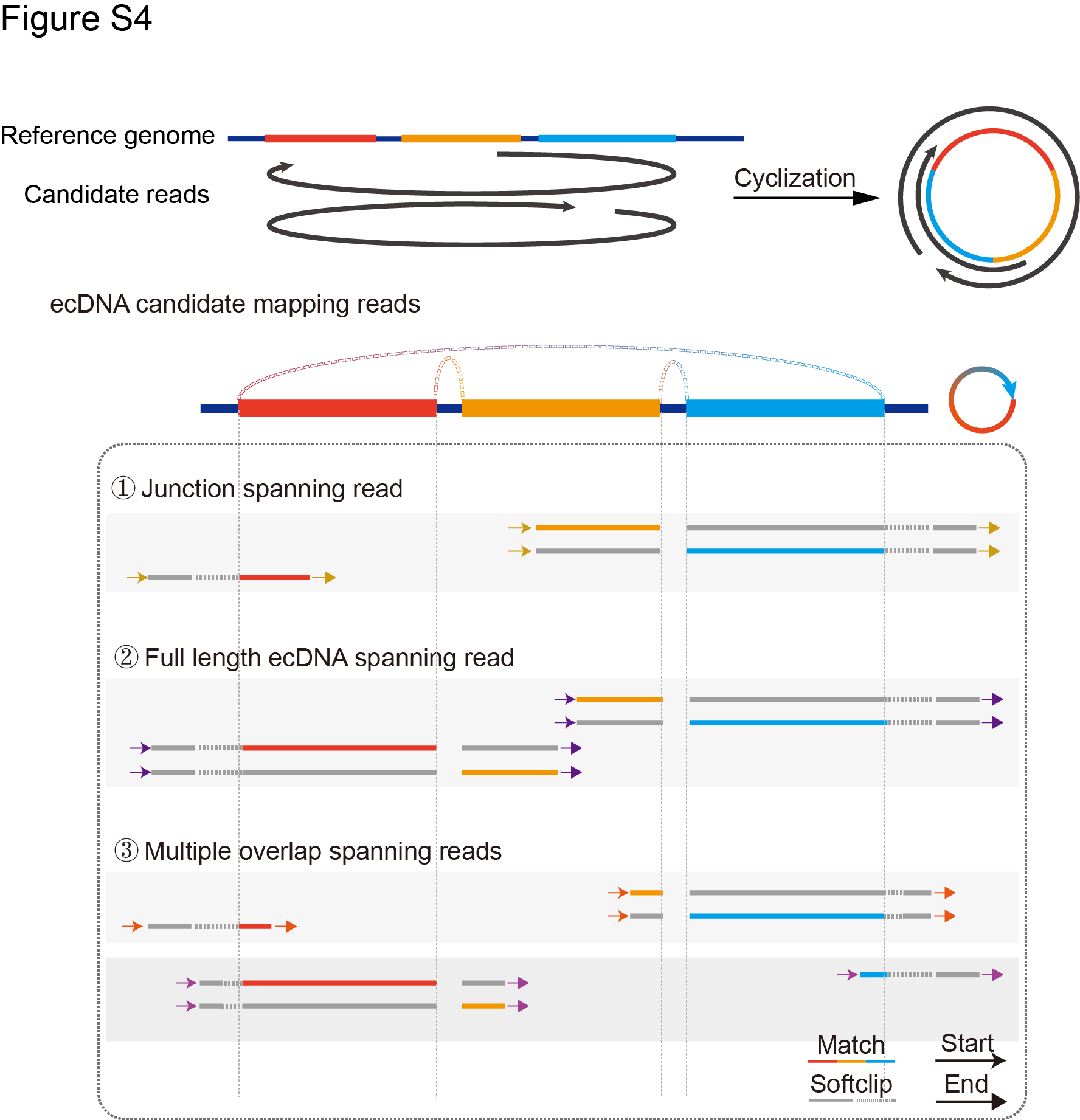


**Figure S4.** The schematic graph of ecDNA and reads mapped to it. How ecDNAs reads are reported in the mapping bam file is shown in the bottom.


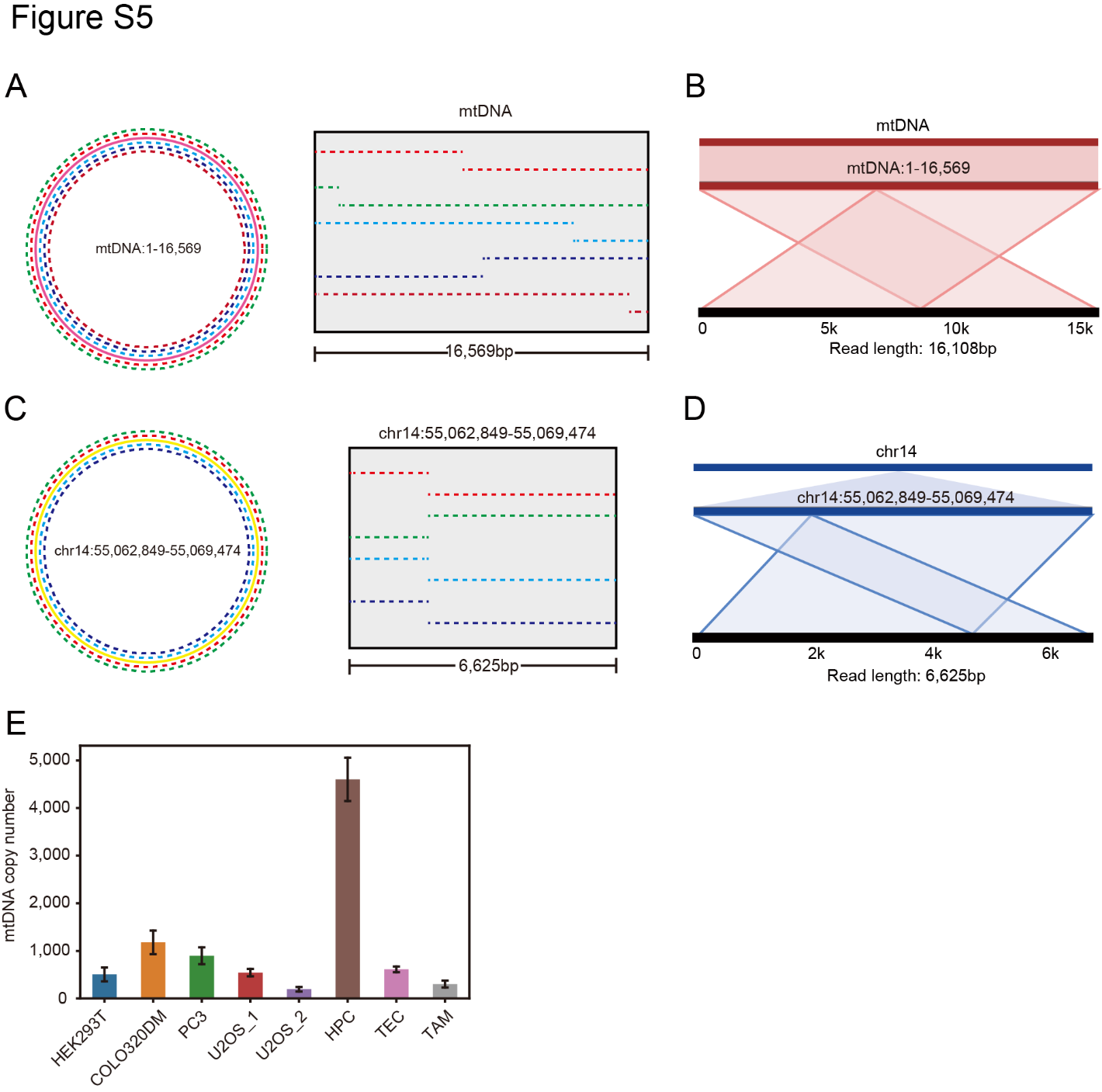


**Figure S5. Analysis on ecDNAs and mtDNA**

(A) Examples of one single sequencing read covering full-length of mtDNA.

(B) One specific read and its mapping to the mtDNA.

(C) Examples of one single sequencing read covering full-length of ecDNA generated from chr14.

(D) One specific read and its mapping to the chr14.

(E) The statistics of mtDNA copy number in different cell types. Data are presented as mean ± SE.


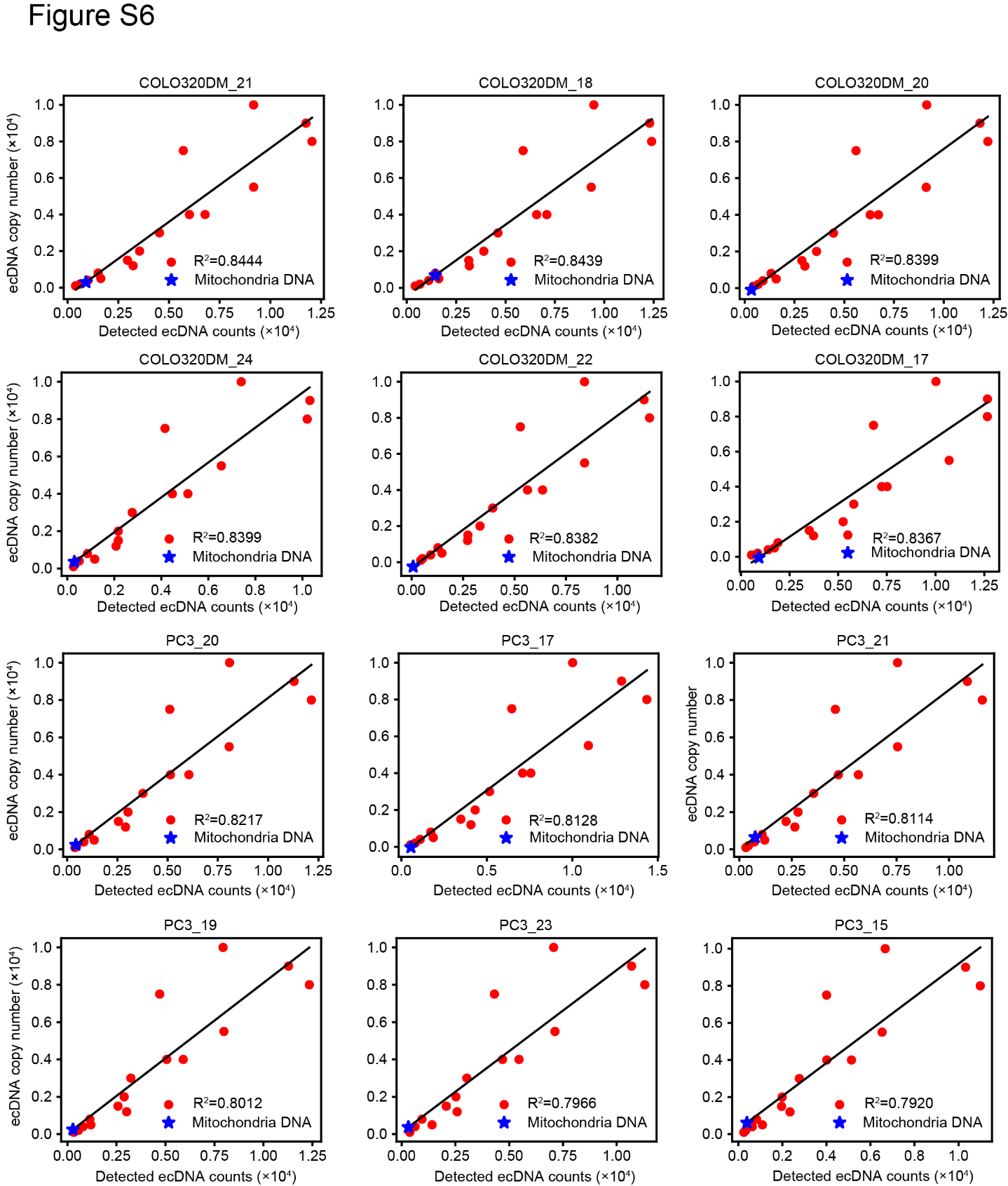


**Figure S6.** Additional examples of correlation analysis between the detected ecDNA counts and the absolute copy numbers for 16 exogenous plasmids (as in Figure 2C).


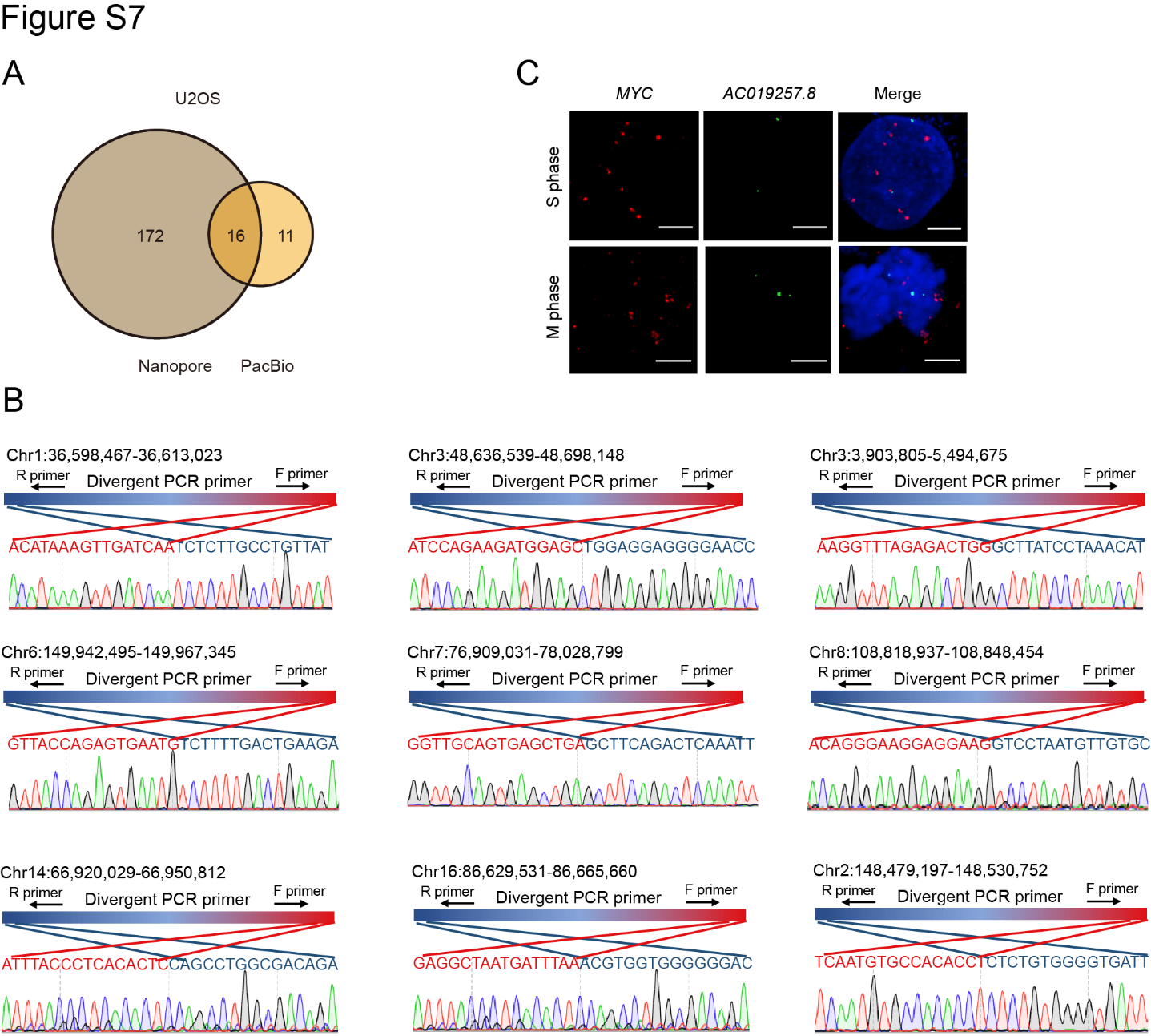


**Figure S7.** Additional analysis on ecDNAs identified by scGTP-seq

(A) Venn diagram describing the overlap of the ecDNAs detected from Nanopore and PacBio sequencing data in U2OS cells.

(B) Sanger sequencing results of the outward PCR products of ecDNA cyclization sites showed in Figure 2E.

(C) Representative 3D-projection images of ecDNA located gene *MYC* and a non-ecDNA located gene *AC019257.8* on chromosome 8 labeled by Oligopaint DNA FISH probes in interphase and metaphase U2OS cell. Red: FISH signals of *MYC* gene. Green: FISH signals of *AC019257.8* gene. Blue: DAPI staining. Scale bars, 5 µm.


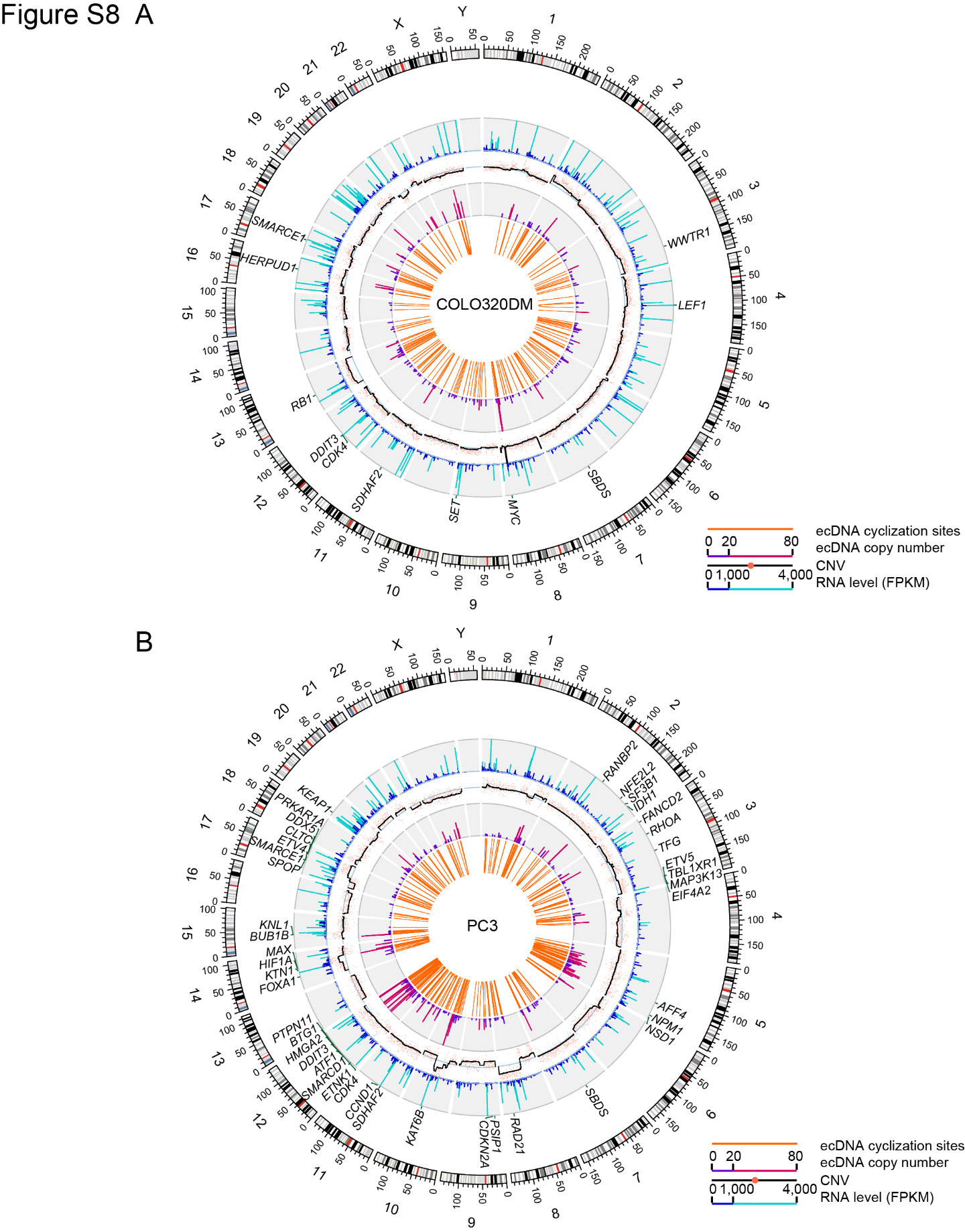


**Figure S8.** Circos plot showing the landscape of ecDNAs in COLO320DM (A) and PC3 (B) cell lines. Circos plot are generated in the same way as in Figure 3A.


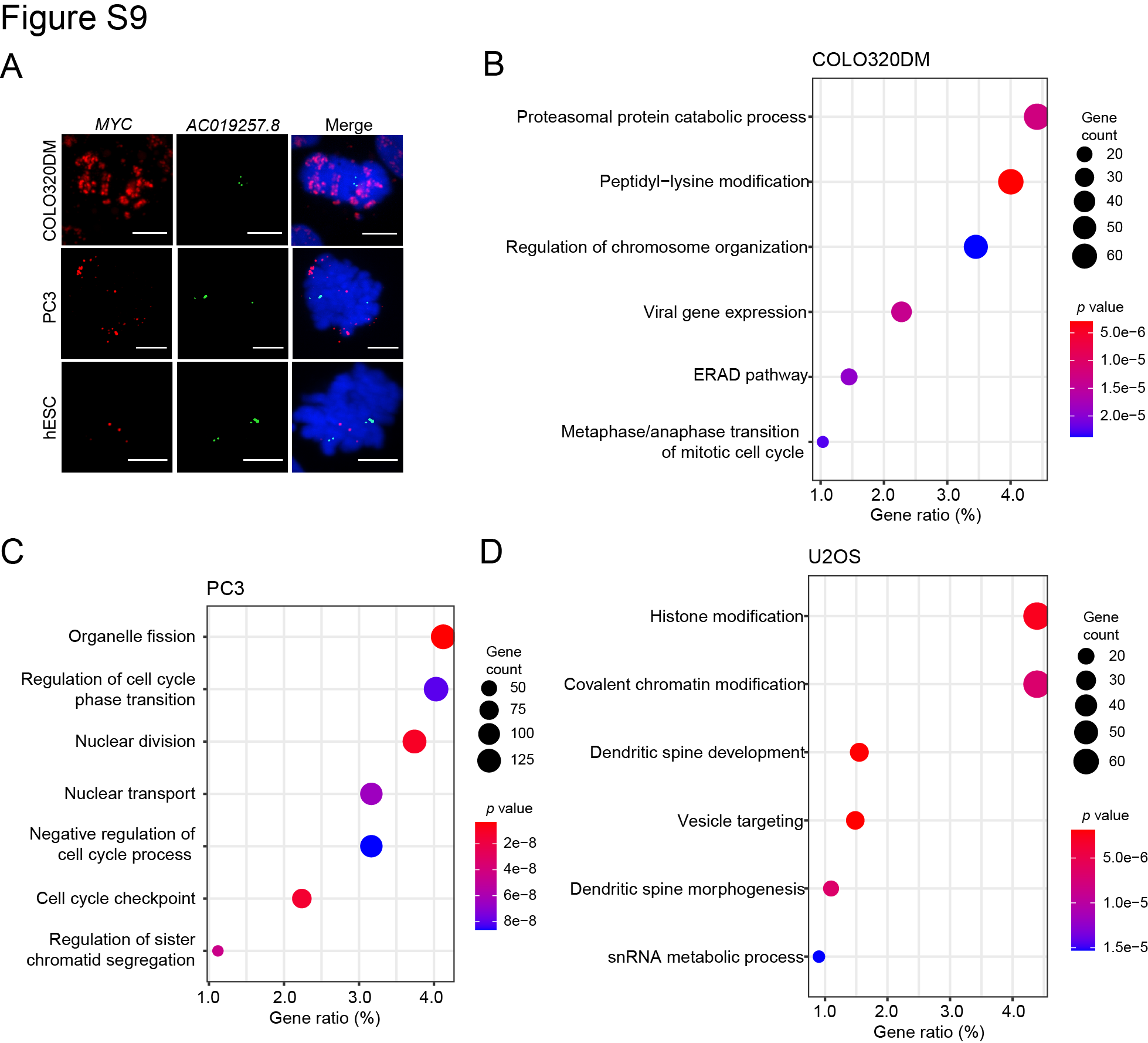


**Figure S9.** ecDNA genes analysis.

(A) Representative metaphase 3D-projection images of ecDNA located gene *MYC* and a non-ecDNA located gene *AC019257.8* on chromosome 8 labeled by Oligopaint DNA FISH probes in three different cell lines. Red: FISH signals of *MYC* gene. Green: FISH signals of *AC019257.8* gene. Blue: DAPI staining. Scale bars, 5 µm.

(B-D) GO terms enriched by ecDNA genes detected in (B) COLO320DM, (C) PC3 and (D) U2OS cell lines respectively.


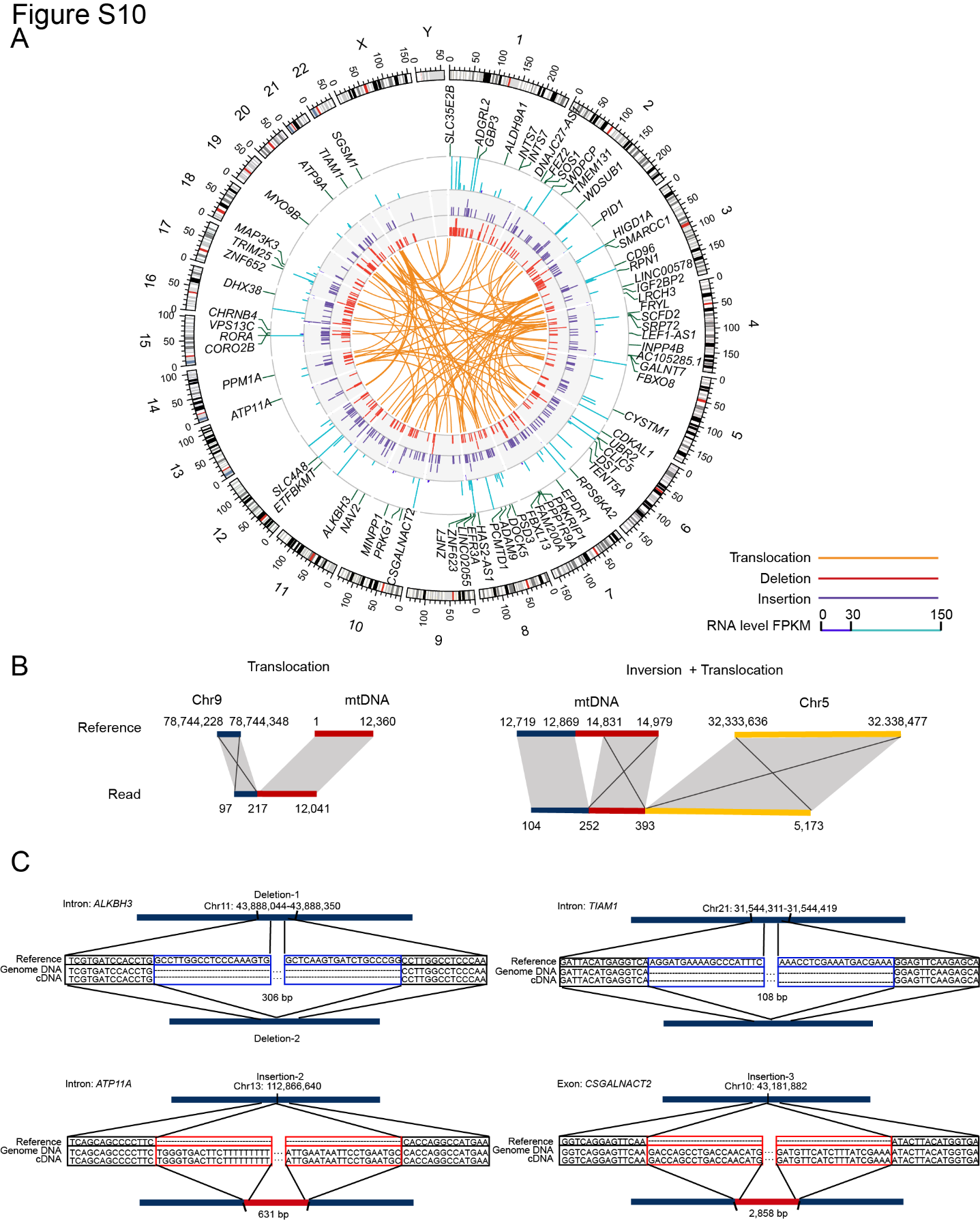


**Figure S10.** SVs analysis

(A) Circos plot showing the landscape of SVs of U2OS cells. Information from inner to outside of the circular map: translocation events (orange, supporting cell number>3), deletions (red), part of insertions (purple), RNA levels (cyan and blue). Genes with SVs transcripts are labeled, including 27 genes with deletion (supporting cell number>2) and 43 genes with insertion (supporting cell number>3).

(B) The insertions and translocation events on mtDNA.

(C) Sanger sequencing results of PCR products from Deletion-1, Deletion-2, Insertion-2 and Insertion-3, as shown in Figure 4F.


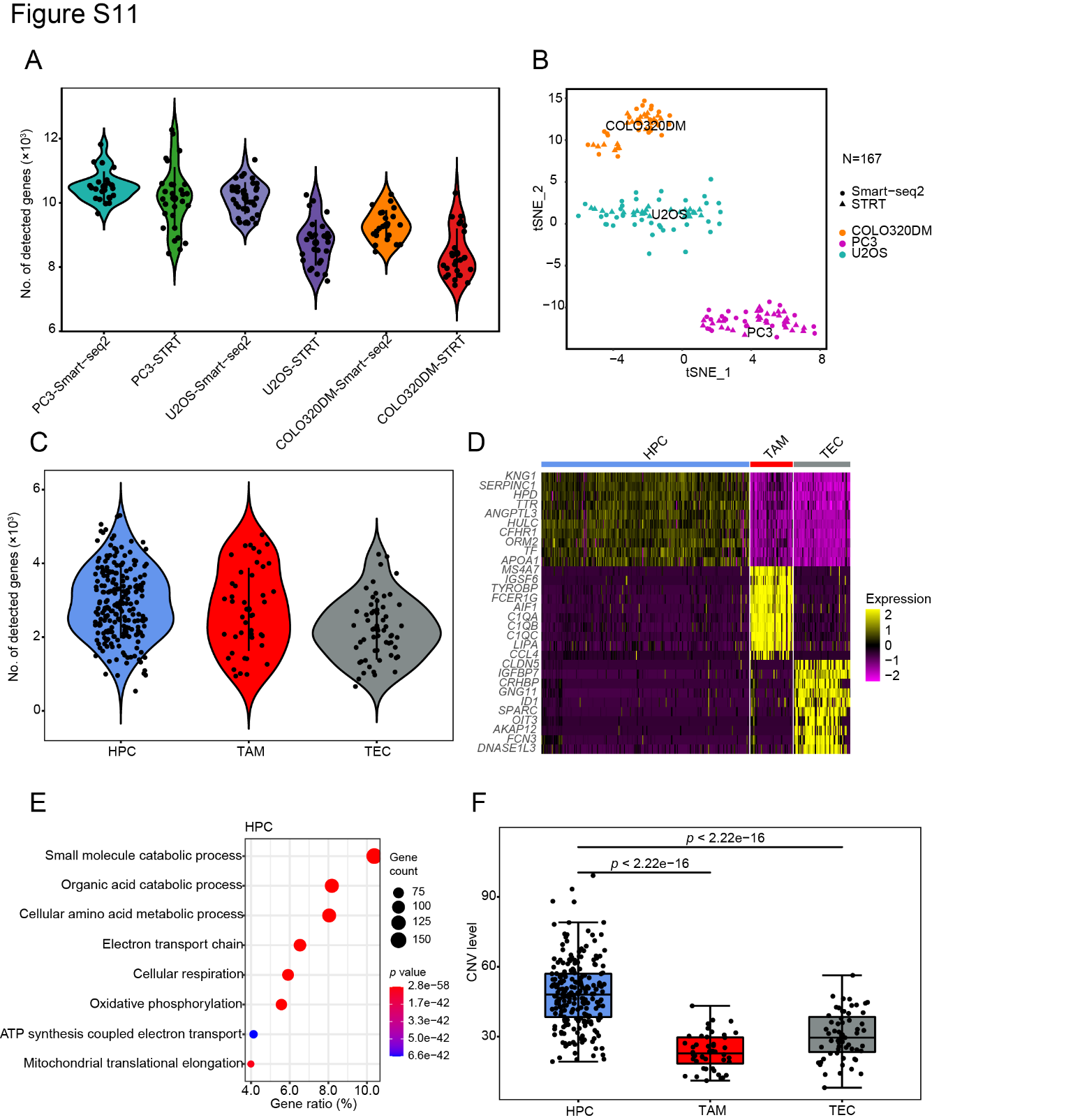


**Figure S11.** Gene expression analysis of HCC.

(A) Violin plot showing number of genes detected in each single cell belonging to three different cancer cell types and measured by Smart-seq2 and STRT techniques separately.

(B) tSNE plot showing cells from different groups as shown in (A).

(C) Violin plot showing number of genes detected in each single cell from three cell types in the HCC sample.

(D) Heatmap showing differentially expressed marker genes in different cell types.

(E) GO terms for differentially expressed genes in HPC cells.

(F) Boxplot displaying copy number variants in different cell types from RNA sequencing data. *p* values are calculated using two-sided Wilcoxon-Mann-Whitney test.


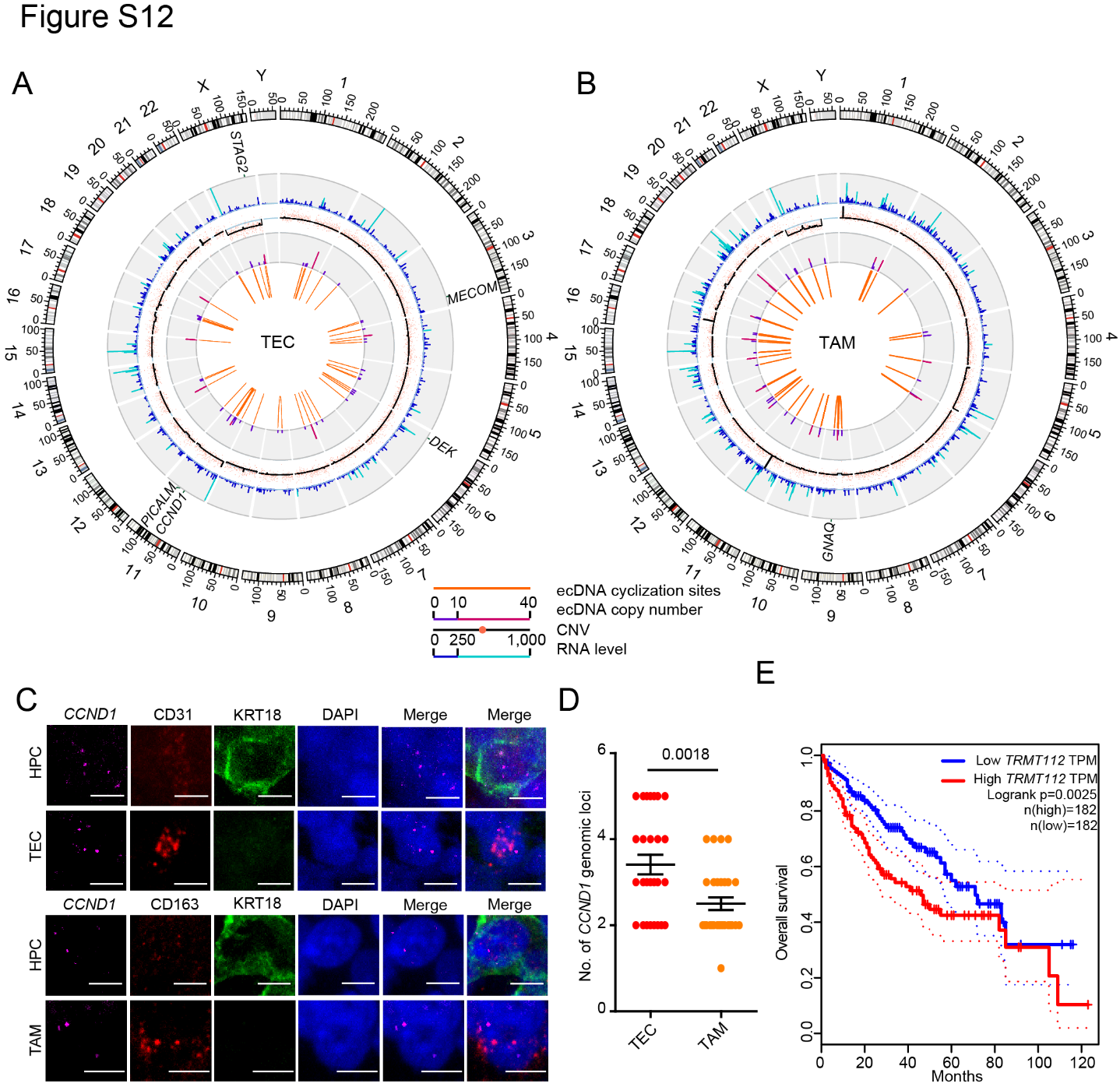


**Figure S12.** Application of scGTP-seq in a clinical HCC sample

(A) – (B) Circos plots showing the landscape of ecDNAs in TECs (A) and (B) TAMs. Circos plots were generated as in Figure 5D.

(C) Immuno-FISH images of ecDNA located gene *CCND1* and marker proteins of HPC (KRT18), TEC (CD31) and TAM (CD163) in HCC tissue slices. Purple: FISH signals of *CCND1* gene. Red: immuno-fluorescent signals of CD31/CD163. Green: immuno-fluorescent signals of KRT18. Blue: DAPI staining. The maximum intensity projections of nuclear Z stacks are displayed. Scale bars, 5 µm.

(D) Quantification of *CCND1* gene copy number from Immuno-FISH images in TECs (N=27) and TAMs (N=28). Each dot represents a nucleus. Black lines present mean ± SE. *p* values are calculated using two-tailed Student’s *t*-test.

(E) Overall survival curve analysis for ecDNA gene *TRMT112* from the HCC patients.


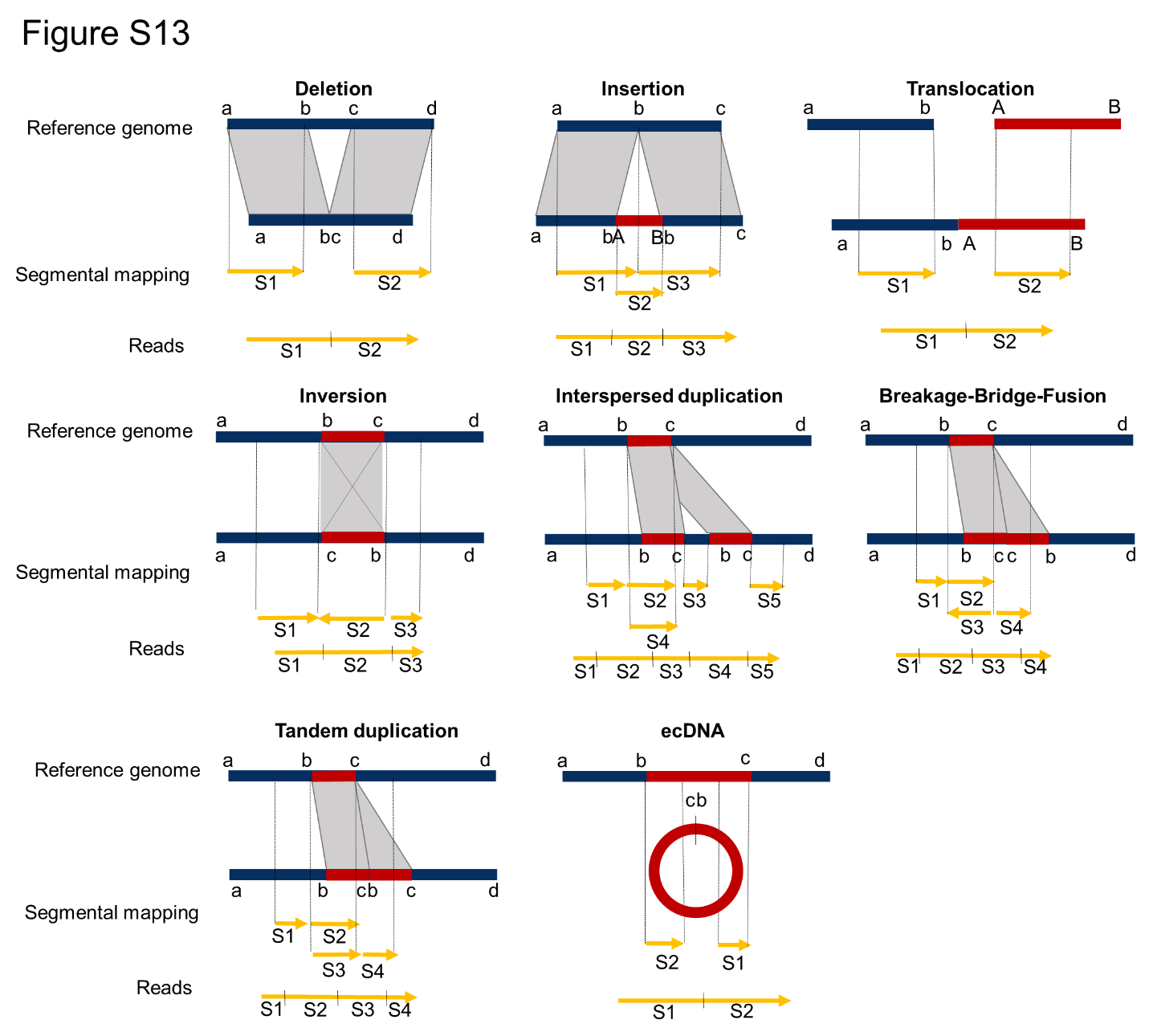


**Figure S13.** The schematic graph of the reads covering the breakpoints of different SVs.
